# Development and Evaluation of the Usefulness, Usability, and Feasibility of iNNOV Breast Cancer: Mixed Methods Study

**DOI:** 10.2196/33550

**Published:** 2022-02-15

**Authors:** Cristina Mendes-Santos, Francisco Nunes, Elisabete Weiderpass, Rui Santana, Gerhard Andersson

**Affiliations:** 1 Department of Culture and Society Linköping University Linköping Sweden; 2 Experimental Pathology and Therapeutics Group Portuguese Institute of Oncology Porto Portugal; 3 Public Health Research Centre NOVA National School of Public Health Universidade Nova de Lisboa Lisbon Portugal; 4 Comprehensive Health Research Center Portugal; 5 Fraunhofer Portugal AICOS Porto Portugal; 6 International Agency for Research on Cancer Lyon France; 7 Department of Behavioural Sciences and Learning Linköping University Linköping Sweden; 8 Department of Biomedical and Clinical Sciences Linköping University Linköping Sweden; 9 Department of Clinical Neuroscience Psychiatry Section Karolinska Institutet Stockholm Sweden

**Keywords:** acceptance and commitment therapy, anxiety, breast cancer survivors, cognitive behavioral therapy, depression, digital mental health, e-mental health, user-centered design, internet interventions, usability, mobile phone

## Abstract

**Background:**

Despite the efficacy of psychosocial interventions in minimizing psychosocial morbidity in breast cancer survivors (BCSs), intervention delivery across survivorship is limited by physical, organizational, and attitudinal barriers, which contribute to a mental health care treatment gap in cancer settings.

**Objective:**

The aim of this study is to develop iNNOV Breast Cancer (iNNOVBC), a guided, internet-delivered, individually tailored, acceptance and commitment therapy–influenced cognitive behavioral intervention program aiming to treat mild to moderate anxiety and depression in BCSs as well as to improve fatigue, insomnia, sexual dysfunction, and health-related quality of life in this group. This study also aims to evaluate the usefulness, usability, and preliminary feasibility of iNNOVBC.

**Methods:**

iNNOVBC was developed using a user-centered design approach involving its primary and secondary end users, that is, BCSs (11/24, 46%) and mental health professionals (13/24, 54%). We used mixed methods, namely in-depth semistructured interviews, laboratory-based usability tests, short-term field trials, and surveys, to assess iNNOVBC’s usefulness, usability, and preliminary feasibility among these target users. Descriptive statistics were used to characterize the study sample, evaluate performance data, and assess survey responses. Qualitative data were recorded, transcribed verbatim, and thematically analyzed.

**Results:**

Overall, participants considered iNNOVBC highly useful, with most participants reporting on the pertinence of its scope, the digital format, the relevant content, and the appropriate features. However, various usability issues were identified, and participants suggested that the program should be refined by simplifying navigation paths, using a more dynamic color scheme, including more icons and images, displaying information in different formats and versions, and developing smartphone and tablet versions. In addition, participants suggested that tables should be converted into plain textboxes and data visualization dashboards should be included to facilitate the tracking of progress. The possibility of using iNNOVBC in a flexible manner, tailoring it according to BCSs’ changing needs and along the cancer care continuum, was another suggestion that was identified.

**Conclusions:**

The study results suggest that iNNOVBC is considered useful by both BCSs and mental health professionals, configuring a promising point-of-need solution to bridge the psychological supportive care gap experienced by BCSs across the survivorship trajectory. We believe that our results may be applicable to other similar programs. However, to fulfill their full supportive role, such programs should be comprehensive, highly usable, and tailorable and must adopt a flexible yet integrated structure capable of evolving in accordance with survivors’ changing needs and the cancer continuum.

## Introduction

### Background

Since 2020, breast cancer has been the most diagnosed cancer worldwide and the leading cause of cancer mortality in women. In Portugal, as many as 7041 women are diagnosed per year with breast cancer, and in 2020, a total of 1864 women died owing to the condition [1,2]. Nevertheless, owing to improvements in early diagnosis, tumor molecular characterization, and innovative systemic treatments, breast cancer prognosis has significantly improved across the globe, with 5-year survival rates reaching approximately 90% in high-income countries [3]. In Portugal, the 5-year prevalence of breast cancer was estimated at 27,051 in 2020, making breast cancer survivors (BCSs) the largest group of cancer survivors in the country [1,2].

In spite of a positive prognosis, the survivorship trajectory is frequently characterized by difficulties associated with sequelae of cancer and its treatment and late physical and psychosocial effects that hinder BCSs’ health-related quality of life (HRQoL) [4]. Anxiety, depression [5,6], fear of recurrence [7], fatigue [8], sleeping problems [9], and sexual dysfunction [10,11] are among the most common problems BCSs experience across survivorship and can manifest up to several years after primary treatment completion [12].

In the past decades, several interventions have been developed to minimize psychosocial morbidity in BCSs. Recent meta-analyses demonstrated the efficacy of such interventions in improving a range of psychosocial outcomes [13,14]. Cognitive behavioral therapy (CBT) has been identified as the most effective intervention to treat anxiety and depression in BCSs, often showing significant small-to-moderate treatment effects in patients with these conditions [13,14]. Other psychosocial interventions such as psychoeducational treatments [14], mindfulness-based interventions [15], and acceptance and commitment therapy (ACT) have been tested among BCSs with success as well [16]. ACT, owing to its model of healthy adaptation to difficult circumstances and transdiagnostic approach, has been appointed as particularly useful in addressing the high levels of psychological and medical comorbidities that manifest in cancer populations [17,18]. Regrettably, the delivery of such interventions across the survivorship trajectory is limited owing to distance from health care services, health care system limitations, mental health illiteracy, and attitudinal barriers, all of which contribute to a mental health care treatment gap in cancer settings [19].

Internet interventions—self-help technology-enabled interventions that provide synchronous or asynchronous health-related and mental health–related assistance based on established psychotherapy models [20]—provide an opportunity to fulfill the mental health care gap within oncology and offer BCSs with patient-centered support at a distance. Nevertheless, despite internet interventions’ attested efficacy [21] in treating various mental health conditions and its potential cost-effectiveness [22,23], internet interventions targeting cancer survivors are scant [24]. Although promising effects of such interventions have been documented concerning anxiety, depression [25,26], distress [27], fatigue [28], physical activity [29], symptom management [30], insomnia [31,32], sexual dysfunction [33], and quality of life [34,35], the overall benefit of such interventions for BCSs is still unclear. Most studies in this domain report on dissimilar interventions or present high methodological heterogeneity, which makes their comparison difficult and inconsistent [30,36]. Moreover, interventions’ design processes are rarely reported, and the absence of evidence-based reasoning behind its development [36] contributes to a research-practice gap in the internet interventions domain, wherein evidenced-based treatments struggle to be adopted in routine care [37]. Another cause for the low uptake of internet interventions in clinical settings is the peripheral position end users are often referred to during development [38]. Intervention programs are frequently planned by neglecting end users’ perspective (eg, individual’s goals, needs, skills, and contexts) and researchers often fail to involve end users in the development process [37,38]. This lack of human-centeredness in the development partly explains the high attrition rates and poor engagement often reported in clinical trials and configures a limitation that needs to be addressed to effectively impact survivorship supportive care provision [39,40].

The aim of this study is to report on the development, usefulness, usability, and preliminary feasibility of iNNOV Breast Cancer (iNNOVBC), a guided, internet-delivered, individually tailored, ACT-influenced CBT program developed to treat mild to moderate anxiety and depression in BCSs, as well as to improve fatigue, insomnia, sexual dysfunction, and HRQoL in this group. Besides informing iNNOVBC’s further development and refinement, the contribution of this paper resides in the description of mental health professionals’ (MHPs’) and BCSs’ perspectives on the use of digital technology to support cancer survivors and the design implications that arise from considering these.

### iNNOVBC Overview

**Figure 1:**
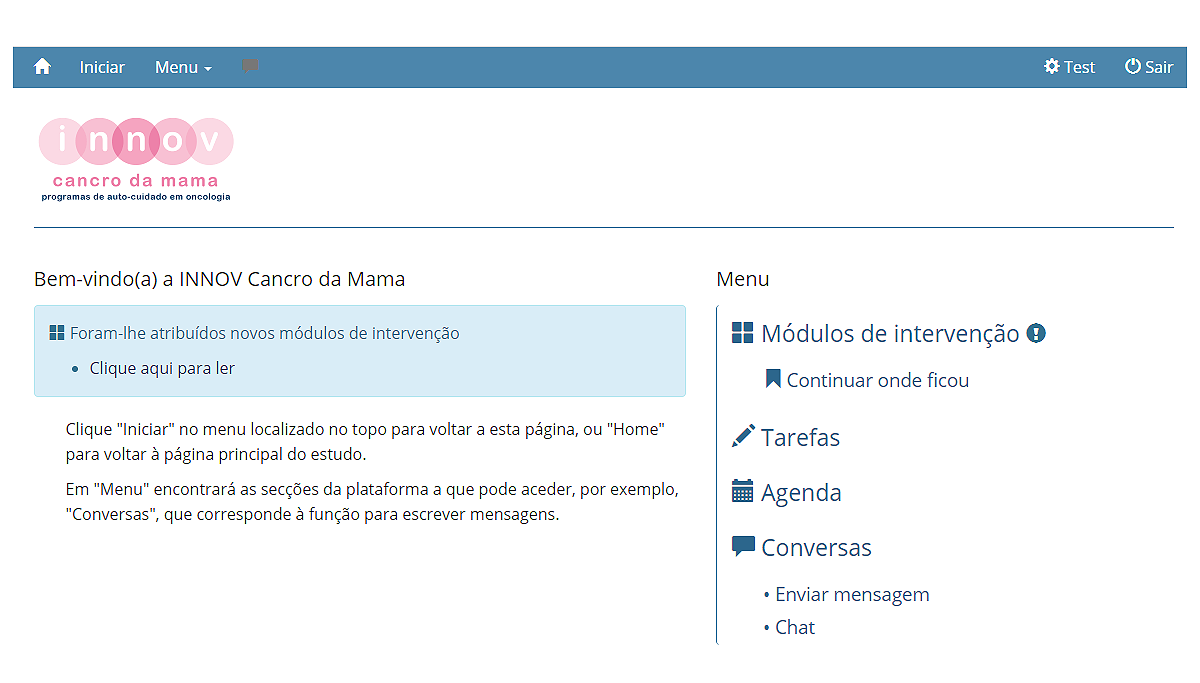
The landing page of the iNNOV Breast Cancer program.

**Figure 2:**
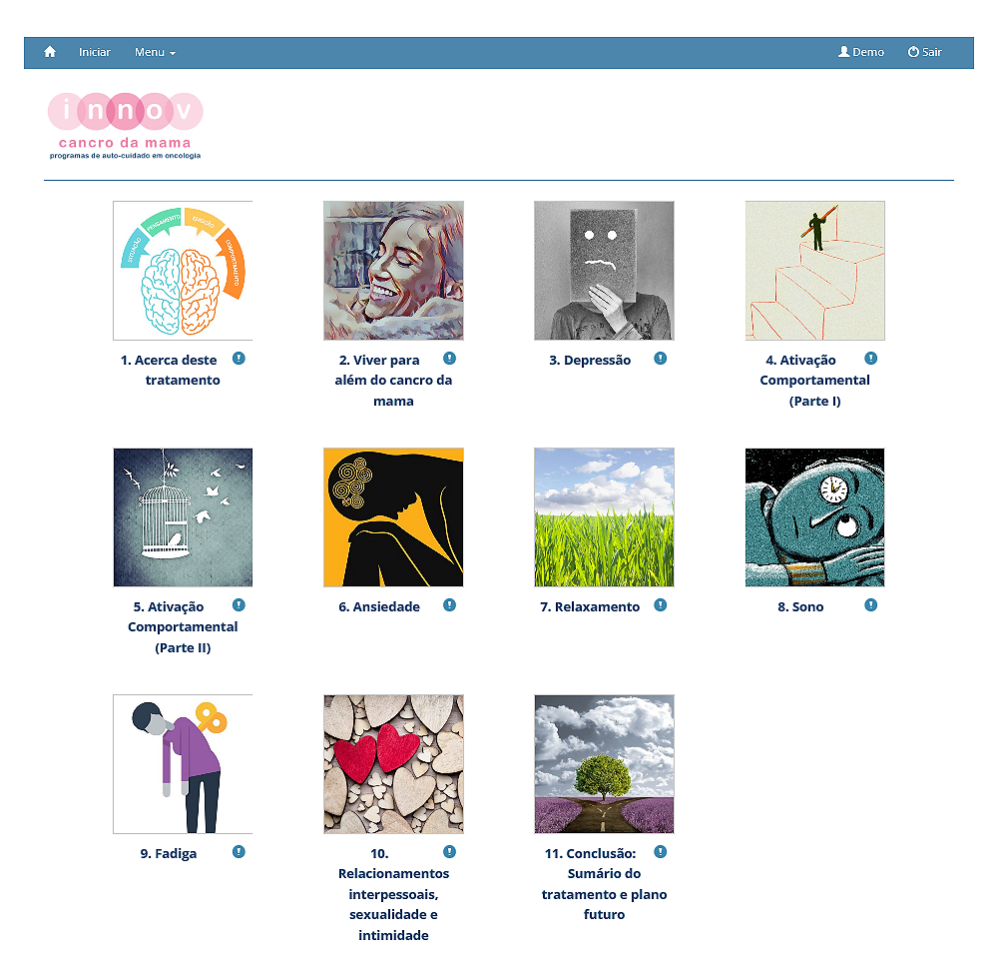
The introductory and 10 treatment modules of the iNNOV Breast Cancer program.

iNNOVBC (Figures 1 and 2) is a guided, internet-delivered, individually tailored, ACT-influenced CBT program and was developed with a user-centered design approach [37]. The program was created to address the psychosocial needs of BCSs that were previously identified via literature review, namely, anxiety, depression, fatigue, insomnia, sexual dysfunction, and HRQoL. The intervention structure and content build up on prior CBT- and ACT-inspired interventions developed by the Department of Behavioural Sciences and Learning at Linköping University, targeting other populations [41-45]. The applicable previously available content was translated from Swedish and English to Portuguese and reviewed by external experts (ie, an oncologist, a nurse, a psychologist, and a BCS). Additional content was developed based on peer-reviewed sources [46-50], as well as mixed methods research conducted by the research team and involving iNNOVBC’s primary and secondary end users [51,52].

iNNOVBC is composed of 10 treatment modules (Multimedia Appendix 1), namely, five mandatory modules (living with breast cancer and beyond, depression, anxiety, relaxation, and key points summary, and planning for the future) and five optional modules (behavioral activation parts I and II, sleep fatigue, and interpersonal relationships, sex, and intimacy), to be completed in 10 weeks. An introductory module provides general information about the program. Each module is designed to be completed in approximately 60 minutes and includes written text, images, videos, audio files, quizzes, ACT- and CBT-based exercises, homework assignments, and respective worksheets. The program adopts a transdiagnostic structure, featuring psychoeducation, acceptance, cognitive defusion, connecting with values, committed action, exposure, behavioral activation, and relaxation as central components. Sleep management, energy conservation, problem solving, and sensate focusing techniques are complementary components of the program. To guarantee optimal use of the program, the study intervention was developed according to the following persuasive system principles categorized by Oinas-Kukkonen and Harjumaa [53]: responsiveness, tunneling, tailoring, personalization, reminders, and professional support.

At the onset of the intervention, BCSs using the program should tailor their treatment with the support of their assigned therapist and according to their baseline assessment and preferences. Once they reach an agreement, the selected modules should be prescribed and made available weekly to the BCSs. Then, BCSs are prompted to complete the modules in approximately 1 week. Within 24 hours of module completion, the therapists assess the BCSs’ progress based on the reported outcomes and determine whether they should proceed to the next module. When a new module is made available, BCSs receive an email notifying them. If not, therapists should instruct them on what needs to be completed to be able to advance to the next module. Integrated 2-way communication features such as email, chat, SMS text messaging, and videoconference support the intervention. The program is delivered via iTerapi, a web-based treatment platform developed at Linköping University [54].

## Methods

### Study Design

**Figure 3:**
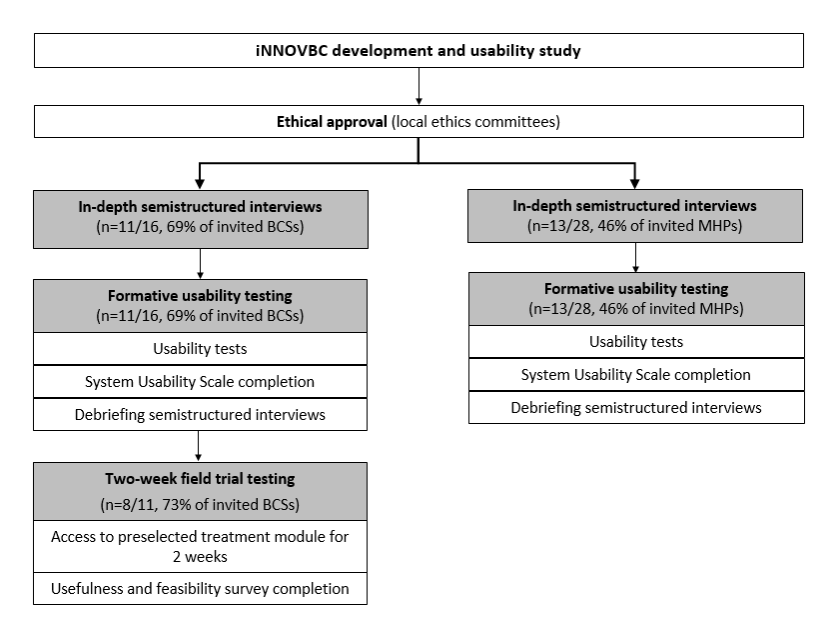
Study design. BCS: breast cancer survivor; iNNOVBC: iNNOV Breast Cancer; MHP: mental health professional.

Mixed methods (Figure 3), namely in-depth semistructured interviews, usability tests, short-term field trials, and surveys, were combined to fulfill the following specific goals: (1) evaluate iNNOVBC’s usefulness, (2) assess iNNOVBC’s usability, and (3) explore iNNOVBC’s perceived feasibility and acceptability among its target users. The study was approved by the ethical committees of Instituto Português de Oncologia do Porto, Francisco Gentil, EPE; Centro Hospitalar Universitário do Porto; Centro Hospitalar São João; Unidade Local de saúde–Matosinhos; Hospital CUF Porto; Ordem dos Psicólogos Portugueses; and the Portuguese Data Protection Committee (approval number: 10727/2017). Written informed consent was obtained from all participants.

### Sampling and Recruitment

The study targeted BCSs and MHPs, who are iNNOVBC’s primary and secondary end users. Eligibility criteria for BCSs consisted of women, aged >18 years, with a history of histologically confirmed breast cancer, who had completed primary adjuvant treatment (except hormonal therapy), and were capable of reading and writing in Portuguese. MHPs were registered psychologists or psychiatrists, capable of reading and writing in Portuguese. A nonprobabilistic sample of BCSs and MHPs was recruited following referrals from the researchers and professionals working at treatment centers in Porto (Portugal) and via snowball sampling. Participants were purposively sampled for diversity in age, academic degree, and digital technology proficiency. A total of 28 MHPs and 16 BCSs were invited to participate in the study in person, via email, or by telephone. Of the invited individuals, 54% (15/28) of the MHPs and 88% (14/16) of the BCSs agreed to participate. Meaning saturation was used as a stopping criterion, which meant that new participants would not be enrolled once novel fieldwork insights stopped changing analysis significantly [55]. Data collection ended after 87% (13/15) of the MHPs and 69% (11/16) of the BCSs completed the research protocol, as meaning saturation was reached during the last interviews and usability tests.

### Data Collection Procedures

Data were collected between November 2019 and February 2020 (ie, before the COVID-19 pandemic). Up to 2 to 3 interviewers or moderators (CMS, Ana Alves, and Elsa Oliveira) participated in data collection. The research protocol included an exploratory semistructured interview (N=24; ie, 11/24, 46% BCSs and 13/24, 54% MHPs; Multimedia Appendix 2), a laboratory-based usability test (Multimedia Appendix 3), followed by the completion of a self-reported usability survey [56] (N=24; ie, 11/24, 46% BCSs, and 13/24, 54% MHPs), and a debriefing post usability test interview (N=24; ie, 11/24, 46% BCSs, and 13/24, 54% MHPs; Multimedia Appendix 4). The exploratory interview aimed to investigate the usefulness of iNNOVBC and gather requirements for further refinement of the program. The usability test assessed the participants’ performance while executing a series of representative predefined tasks on distinct parts of the platform. The think-aloud protocol was implemented to enable participants to voice their thoughts and issues [57]. The debriefing interview focused on participants’ experience with using the platform, issues hindering their experience, and changes to be performed to achieve better effectiveness, efficiency, and satisfaction with the program. These activities occurred in person at Fraunhofer Portugal–Centre for Assistive Information and Communication Solutions meeting rooms. Participant BCSs willing to further assess iNNOVBC were invited to participate in an additional 2-week field trial (8/11, 73% BCSs; Multimedia Appendix 5) mimicking the experience of using the program, and to fill out a survey through the platform on the perceived usefulness and feasibility of the program (Multimedia Appendix 6).

Interviews and usability tests were audio and video recorded, respectively. Approximately 36 hours of audio recordings and 9 hours of video recordings were created. The average duration of pretest interviews was 49 minutes (range 26-81 minutes) for MHPs and 74 minutes for BCSs (range 39-106 minutes). Usability tests were completed on average in 20 minutes and followed by 28 minutes (range 10-46 minutes) of debriefing interviews. Interview recordings were transcribed verbatim (CMS and Ana Alves) in parallel to data collection, using oTranscribe (created by Elliot Bentley; a project of the MuckRock Foundation) [58]. Usability tests were assessed using predeveloped observation grids and by registering participants’ voiced remarks. Data were stored in a pseudoanonymized format in a secure password-protected location. 

### Measures

#### Clinical, Sociodemographic, and Internet-Related Characteristics

Background questionnaires tailored to each target group collected clinical, sociodemographic, and internet-related characteristics. BCSs were inquired about age, gender, education level, marital status, occupation, professional status, distance between residence and treatment center, time since diagnosis, type of treatment performed, survivorship status, proficiency using digital technology, and experience in using digital mental health (DMH) programs. MHPs were inquired about age, gender, education level, marital status, occupation, professional status, work context, professional experience (in years), theoretical orientation, proficiency in using digital technology, and experience in using DMH programs.

#### Usefulness

Semistructured interview guides (Multimedia Appendix 2) were developed based on a literature review to assess participants’ perceived usefulness of the program. The interview guides covered the following domains: (1) survivorship main challenges, unmet care needs, and self-care strategies developed to address those challenges and needs; (2) the provision of psychosocial survivorship care to BCSs and the main barriers impacting it; (3) knowledge and use of DMH; and (4) attitudes toward DMH programs aimed at providing survivorship support. In addition, a questionnaire developed to assess the quality of iNNOVBC’s treatment modules was used for this purpose (Multimedia Appendix 6).

#### Usability

We conducted usability tests and analyzed the performance and acceptance of the system. To assess performance, task analysis was conducted and the number of completed tasks, errors, and assistances were recorded in observation grids (Multimedia Appendix 3). Acceptance was measured using the Portuguese version of the System Usability Scale [56], where a score <68 is considered below average. The debriefing interviews also informed about acceptance and contributed to identifying content and design changes to be performed to improve user experience and satisfaction (Multimedia Appendix 4).

#### Feasibility

The preliminary feasibility of the program was assessed via debriefing semistructured interviews and assessment questionnaire in iNNOVBC’s treatment modules (Multimedia Appendix 6). The participant BCSs who used the system at home also provided written comments.

### Analysis

A combination of quantitative and qualitative methods was applied to assess iNNOVBC’s usefulness, usability, and perceived feasibility. Descriptive statistics, namely, counts, percentages, medians, and IQRs, were used to characterize the study sample, evaluate performance data, and assess preference data collected via the System Usability Scale and the treatment modules’ assessment questionnaire. No efficiency metrics were computed because the think-aloud method was applied. Microsoft Excel was used to compute quantitative variables. Qualitative data resulting from the interviews and BCSs’ written comments were transcribed verbatim and analyzed using the thematic analysis method of Braun and Clarke [59]. First, a deductive approach was adopted based on three predetermined high-level themes: usefulness, usability, and feasibility. Subsequently, inductive analysis was performed on the data collected within those themes, and salient subthemes were coded. Initial coding was performed by the first author (CM). Regular discussions were promoted between researchers (CMS and FN) to discuss results and coding trees. Data patterns were then identified and iteratively organized (CMS and FN) until consensus between researchers was achieved and no additional insights were resulting from the analysis of the data. Scrivener software [60] was used to support the coding process.

## Results

### Participants’ Characteristics

The sample comprised 24 participants (Table 1), including 11 (46%) BCSs and 13 (54%) MHPs. The median age of BCSs was 48 years (IQR 14; minimum 32, maximum 68). Most survivors were married (7/11, 64%) and college-educated (7/11, 64%). Approximately 55% (6/11) of the survivors were professionally active. Most BCSs had been treated with surgery (11/11, 100%), chemotherapy (9/11, 82%), radiotherapy (8/11, 73%), and hormonal therapy (9/11, 82%). Considering the survivorship status [61], the sample was heterogeneous, including participants at acute (2/11, 18%), extended (3/11, 27%), and permanent (6/11, 55%) survivorship stages. No participant had previous experience in using DMH programs, but the majority reported medium (4/11, 36%) to strong (5/11, 45%) skills in using digital technology.

The subsample of MHPs was composed of 15% (2/13) psychiatrists and 85% (11/13) clinical psychologists. Most professionals were female (11/13, 85%), and their median age was 35 years (IQR 11; minimum 25, maximum 56). The median of MHPs’ professional experience was 11 years (IQR 11). Moreover, 92% (12/13) of professionals were active, and 30% (4/13) worked in psycho-oncology services. Approximately half of our sample (7/13, 54%) held a CBT orientation. Considering participants’ proficiency in using digital technology, most professionals (11/13, 85%) classified their skills as medium. Approximately half of professionals (7/13, 54%) reported previous experience in using DMH programs.

**Table 1:** Participants’ characteristics (N=24).

Variables			MHP^a^ (n=13)		BCS^b^ (n=11)
**Sex, n (%)**					
	Female	11 (85)		11 (100)	
	Male	2 (15)		0 (0)	
**Age (years), n (%)**					
	23-30	3 (23)		0 (0)	
	31-40	7 (54)		4 (36)	
	41-50	2 (15)		4 (36)	
	51-60	1 (8)		2 (18)	
	61-70	0 (0)		1 (9)	
**Highest academic degree, n (%)**					
	Basic degree (≤9 school years)	0 (0)		2 (18)	
	Secondary degree (12 school years)	0 (0)		2 (18)	
	University degree	13 (100)		7 (64)	
**Self-reported proficiency in using digital technology, n (%)**					
	Poor	1 (8)		2 (18)	
	Medium	11 (85)		4 (36)	
	Strong	1 (8)		5 (45)	
**Self-reported use of digital mental health, n (%)**					
	None	6 (46)		11 (100)	
	Occasional	4 (31)		0 (0)	
	Regular	3 (23)		0 (0)	

^a^MHP: mental health professional.

^b^BCS: breast cancer survivor.

### Usefulness

iNNOVBC’s perceived usefulness was assessed during the interviews conducted before and after the usability tests and by surveying field trial participants on the usefulness and adequacy of the program’s modules. During the field trials, the content of the modules living with breast cancer and beyond (4/11, 36%), anxiety (1/11, 9%), relaxation (1/11, 9%), sleep (1/11, 9%), and fatigue (1/11, 9%) were appraised, with all being rated with 5 (out of 5) stars by BCSs evaluating it. Overall, participants found the program’s approach, content, and features as highly relevant and useful. BCSs classified the content of the modules as very useful (3/8, 36%) or extremely useful (5/8, 63%) and very adequate (3/8, 36%) or extremely adequate (4/8, 50%) in addressing their difficulties and needs. Only 13% (1/8) of the participants reported that the relaxation module was neither adequate nor inadequate to her needs. All survivors (8/8, 100%) reported that they would recommend the modules to a friend or family member going through the same condition.

Considering the role iNNOVBC could play in BCSs supportive care, both survivors and professionals considered iNNOVBC could have a significant impact in supporting BCSs throughout the cancer continuum:

There were a lot of doubts, fevers, discomforts, disquietudes...there still are...and if I had this type of resource, I could have used it instead of googling or looking into patient groups, for some kind of answer...Because you do feel the need to go to forums and ask, does anyone feel like this? What have you done? And if there were a platform like this, the type of support provided would be different, more credible and appropriate.

BCS11

Similar to BCS11, most participants valued the possibility of accessing “trustworthy” (BCS6) self-care information provided “anytime, anywhere” (BCS7) via iNNOVBC. The interviewed BCSs had searched on the web for information on treatments’ adverse events, practical strategies, and emotional challenges they were faced with, but their searches were lengthy and required them to assess the quality of information they were presented with. Both BCSs and MHPs considered that having “easy access to evidence-based ready to use psycho-educational content” (MHP10) could help survivors better manage their condition and problems.

According to the participants, the survivorship trajectory is characterized by many biopsychosocial challenges that deeply impact survivors’ well-being. Several participants described the pervasive and long-lasting impact that treatment’s adverse events or sequelae had on their physical and mental health, underlining the importance of receiving accurate and comprehensive information about it. Effects such as alopecia, onycholysis, pain, menopausal symptoms, fatigue, cardiotoxicity, lymphedema, osteoporosis, infertility, and memory loss were often discussed by interviewees as key information points to address in a nuanced and dynamic manner. During the course of treatment, various survivors received flyers and booklets about treatments’ adverse effects, but most complained about their unappealing design and low intelligibility and focus on the active treatment stage, therefore failing to provide a continuous perspective on its management after treatment completion. The fact that iNNOVBC approached many of those themes “in a chronological way” (BCS1) was appreciated by various BCSs because it enabled them to prepare and cope with it in the long term.

The impact of cancer and its treatments on survivors’ emotional health was another matter of concern. Both MHPs and BCSs underlined the importance of providing psychological support to BCSs throughout the cancer continuum, particularly during the transition from active treatment to follow-up care:

Getting psychological support is very important at all stages...at the diagnosis, during treatment and after treatment...People are not aware of this, they tend to say “Ah, that’s over now!” and expect us to return to whom we used to be...But that’s not possible...you still need support. This feels almost like...a post-traumatic situation...and by saying that it’s almost like they’re taking away your legitimacy to feel the pain, so it can feel very isolating to cross that path.

BCS2

It’s an abrupt shift from being extremely cared for, from having that unconditional support during chemo to stopped being cared for...As soon as your hair starts to grow, everyone assumes that everything is fine. But it’s not. I will never be able to say again that I’m fine. I’m not healed, I’m full of fears, anxiety, and sorrow. So...it was extremely hard for me to deal with that because I felt abandoned...people had pulled the rug out from under me.

BCS7

Similar to BCS2 and BCS7, various participants discussed the experience of feeling in distress and unsupported after finishing the primary treatment. Problems such as “dealing with low mood” (BCS11), “feeling anxious all the time” (BCS2), “fearing that cancer had returned” (BCS8), or fear of “dying” (BCS9) were common, often impacting BCSs’ sleep and interpersonal relationships. Difficulties in adapting to a “scarred body image” (BCS5) were also prevalent in interviewees’ narratives and various participants reported on the profound impact it had on their self-esteem, sexuality, and intimate relationships:

Losing my breast shook my self-esteem, it shook everything...it’s hard to live without it, I’m a woman. I...I’m incapable of being naked in front of my husband. I feel embarrassed...I’m not able to hug him anymore. I’m afraid he feels that it’s not my breast, that it isn’t real. I sleep...without the prosthesis, but I always try to sleep with my back to him and when I realize I’m facing him, I turn over right away...I’m afraid that if he sees me, he might lose his interest in me, that he stops desiring me. ...We still have sex but it’s not the same. I’m always afraid that he touches where he’s not supposed to...I do not lean against him...I avoid his touch. It’s difficult and...I know that there are women that deal with this well, but I...I do not.

BCS9

Similar to BCS9, various participants talked about not being able to adapt to their changed self, feeling like a fake version of themselves, incapable of restoring lost intimacies, and isolated by the “tabu cancer had become” (BCS2) in their inner circles. Coping with such problems was highly demanding, and many struggled to discuss such topics with friends, family, and professionals owing to their sensitive nature. Thus, many interviewees appreciated the “assertiveness of the themes” (BCS6) explored at iNNOVBC and the possibility of receiving professional support at a distance or without face-to-face contact:

It’s important to have support but it’s uncomfortable to talk about these issues face-to-face...Maybe through this platform, it could be easier...I believe I could feel more comfortable to ask some questions if I knew this was anonymous, if I didn’t have to identify me...I believe it would be much easier to approach intimate topics via chat or e-mail than in-person.

BCS3

Participants such as BCS3 appreciated the fact that iNNOVBC allowed direct communication between MHPs and BCSs. Both professionals and survivors considered having chat, email, and videoconference communication alternatives to be helpful. By offering synchronous and asynchronous communication channels associated with different degrees of exposure, participants considered that iNNOVBC “could promote survivors’ self-disclosure” (MHP6) and facilitate the “timely discussion” (MHP4) of sensitive topics, ultimately, “benefiting the therapeutic process” (MHP12). They also saw advantages in the possibility of automatically sending psychoeducational content and scheduling and notifying BCSs about tasks and questionnaires to be completed along the implementation of the program:

The functionalities are well thought and the fact that everything is integrated is awesome...this combines a modular treatment approach with videoconference and chat...it allows you to take notes...and the instruments are embedded...That’s awesome and exactly what I need in my practice!

MHP5

The fact that iNNOVBC combines evidence-based content with communication, monitoring, and documentation features was praised by various professionals. The interviewed MHPs considered that it could reduce the time and effort they need to invest in finding, preparing, and assessing materials, enabling them to work more efficiently. iNNOVBC was also considered useful to promote “continuity of the relationship established between the therapist and the client” (MHP7) beyond appointments. Most importantly, by doing so, iNNOVBC could configure a point-of-need service that could support BCSs along the survivorship trajectory. Nevertheless, it is important to say that iNNOVBC’s usefulness could be compromised by its “lack of intuitiveness” (MHP9).

### Usability

Both the quantitative and qualitative data collected during the usability tests and interviews identified design and functionality issues that could be improved to enable more effective and efficient use of iNNOVBC and increase users’ satisfaction with the program. In general, participants’ effectiveness, as measured by completion rates, was high in both groups (range 69.2-100; Tables 2 and 3), but the System Usability Scale median score was 65 (IQR 35) for BCSs and 47.5 (IQR 25) for MHPs, which may be considered below average in terms of usability. These results are aligned with the usability issues identified during the usability tests.

**Table 2:** MHP^a^ laboratory-based usability test results.

Task groups and tasks		Task performance, %	Error, mean (SD)	Assistances, mean (SD)	
**A: Log-in**					
	1. Log in to the iNNOVBC^b^	100	0.2 (0.4)	0.1 (0.3)	
**B: Notifications**					
	2. Check notifications	84.6	0.8 (1.2)	0.4 (0.6)	
	3. Comply with the notifications’ instructions by accessing the patient’s file	100	2.3 (2.6)	0.7 (0.8)	
**C: Treatment prescription**					
	4. Click on the patient’s link or search for the patient	100	3.2 (5.1)	0.7 (1.1)	
	5. Check the modules prescribed to the patient	100	1.1 (1.2)	0.2 (0.4)	
	6. Assign the sleep module to the patient	100	0.6 (1.1)	0.1 (0.3)	
	7. Send a notification to the patient	69.2	0.4 (0.7)	0.3 (0.8)	
	8. Save the previous procedure	100	0.1 (0.3)	0.2 (0.4)	
	9. Check the available clinical trials	100	3.2 (6.6)	1 (1.3)	
	10. Prescribe iNNOVBC to the patient and schedule the onset of the treatment	100	0.6 (1.3)	0.4 (1.1)	
	11. Save the previous procedure	100	0 (0)	0.1 (0.3)	
**D: Treatment progress assessment**					
	12. Send feedback to the patient about the homework assignment	100	3.8 (5.1)	1.1 (1.2)	
	13. Access the patient’s file	100	0.7 (1.4)	0.4 (0.7)	
	14. Click on questionnaires	100	3.8 (3.0)	1.4 (1.4)	
	15. Check the questionnaires available to prescribe	100	1.6 (3.4)	0.4 (0.8)	
	16. Assign the weekly questionnaire to the patient	100	1.5 (2.4)	0.6 (0.8)	
	17. Save the previous procedure	100	0 (0)	0 (0)	
**E: Conversations**					
	18. Write an email to the patient	100	1.6 (2.1)	0.5 (0.6)	
	19. Send the email	100	0 (0)	0 (0)	
	20. Start a chat conversation with the patient	100	1.5 (2.8)	0 (0)	
	21. Start a videoconference appointment with the patient	100	0.1 (0.3)	0.1 (0.4)	
	22. Update the patient's clinical diary	100	4.4 (5.2)	0.8 (1.2)	
	23. Schedule the next appointment and set an alarm	100	0.6 (0.8)	0.1 (0.3)	

^a^MHP: mental health professional.

^b^iNNOVBC: iNNOV Breast Cancer.

**Table 3:** BCS^a^ laboratory-based usability test results.

Task groups and tasks			Task performance, %		Error, mean (SD)		Assistances, mean (SD)
**A: Log-in**							
	1. Log in to iNNOVBC^b^	100		0.6 (0.8)		0.5 (0.7)	
**B: Notifications**							
	2. Check notifications	81.8		1.3 (1.6)		1.7 (2.5)	
	3. Read the therapist’s message	100		0.5 (1.2)		0.4 (0.8)	
**C: Treatment content management**							
	4. Access treatment modules	100		1.9 (2.6)		2.4 (1.8)	
	5. Access the relaxation module	100		0 (0)		0.4 (1.1)	
	6. Open the deep muscle relaxation page and expand the *how to practice* text	100		2 (1.9)		2 (1.5)	
	7. Play the recorded relaxation session	100		0.5 (0.9)		0.7 (1.4)	
	8. Download the relaxation session	100		0.1 (0.3)		0.4 (1.1)	
	9. Print the current page	100		0.2 (0.4)		0.6 (0.9)	
**D: Worksheet completion**							
	10. Return to the modules	100		0.5 (1.4)		0.9 (2.3)	
	11. Access page 8 of the anxiety module	100		0.1 (0.3)		0.1 (0.3)	
	12. Complete and save anxiety ladder exercise	100		0.3 (0.6)		0.4 (0.9)	
	13. Access the sleep diary	100		0 (0)		0 (0)	
	14. Complete the sleep diary	100		0.5 (1.2)		1.3 (2.5)	
**E: Communicating with therapists**							
	15. Send an email to the therapist	100		0.5 (0.8)		0.6 (0.9)	
	16. Start a chat conversation with the therapist	100		1.1 (1.3)		0.2 (0.6)	
	17. Start a videoconference appointment with the therapist	100		0 (0)		0.5 (1.2)	
**F: Scheduling tasks**							
	18. Schedule a new task and set an alarm	100		0.9 (0.7)		0.8 (1.7)	

^a^BCS: breast cancer survivor.

^b^iNNOVBC: iNNOV Breast Cancer.

According to the classification by Zahabi et al [62], the issues identified during the usability tests (Multimedia Appendix 7) were mostly related to inefficient interaction (12/43, 28%), ineffective information presentation (9/43, 21%), cognitive overload (7/43, 16%), ineffective use of language (6/43, 14%), and lack of naturalness (ie, lack of familiarity or matching between users’ usual workflow and the system; 6/43, 14%). Issues related to consistency (5/43, 12%), feedback (3/43, 7%), customizability and flexibility (3/43, 7%), and error prevention (2/43, 5%) were also identified, although less frequently.

Throughout the usability tests and posttest interviews, participants commented on the importance of making iNNOVBC’s information architecture clearer to facilitate navigation. Various participants verbalized difficulties in understanding how the information and features made available on the platform were hierarchized and could be managed, thus requiring a simplification of navigation paths:

I was always questioning what was for the therapist and what was for the patient...What resources were available to me or them...What was available to prescribe and what was already prescribed...So...I think this should be made clearer.

MHP6

I was a little bit lost because I couldn't find the way...If it were like Pinterest...or facetime, I believe it would be more accessible...[because] All the little windows appear right away, and the images are clear and appealing to me.

BCS4

Similar to BCS4, various participants considered changes to iNNOVBC’s user interface could be performed. Some participants believed the program could be redesigned to display “a unique dashboard with all key features available at login” (BCS8), whereas other interviewees suggested that a tour providing an overview of the platform or adding labels and preview options to most used functionalities would suffice. Furthermore, the use of familiar and interactive design was also appointed as a strategy that could facilitate navigation:

I found it a little monotonous, everything looked the same...and that confused me. Maybe using more colour and highlighting some parts...it would be easier to understand how the materials are organized....

BCS6

Similar to BCS6, various participants expected to find a more appealing and dynamic color scheme, as well as greater use of icons, images, and shortcuts. Despite the perceived adequacy of the content materials, the presentation of long text, sometimes displayed along various binders and features, was considered problematic. Various participants had to do a strenuous effort to screen through the available information and complete the usability test tasks. As a result, participants often became frustrated and some verbalized feeling discouraged to use the program:

I find the anxiety module content very clear, easy to read and very interesting for anxiety sufferers...but the program, in terms of presentation, requires some improvements because sometimes the information displayed is too much.

BCS2

Not only from the therapists’ perspective but also from the patients' there are things that should be simplified so that they do not experience the same frustration I felt because they would give up on using the program...Instead of making it easier, I am afraid they would have to press so many buttons, that they would not understand what is expected from them, and they would give up...ending up not sending anything.

MHP3

Corroborating MHP3, various participants mentioned that simplifications to the provided materials should be performed. Participants suggested organizing the information more concisely, in accordance with single themes, providing hyperlinks to additional information whenever necessary. Participant BCSs also underlined the importance of simplifying the exercise worksheets owing to difficulties in handling tables (eg, sleep and symptom diaries), suggesting that information should be entered using plain textboxes:

It should have an option to enter the date and then the system would label it as register 1, 2, 3, etc...automatically. This shouldn't be a table, it should be a simple field to complete and then the psychologists would see the table. If it's important to the patient to see the progress, a different graphical approach should be used...considering that there are a lot of items to be filled in the platform, I believe having a summary, and overview...that helped me realize my progress...maybe presented as a graph, not only in writing...and something beautiful to see...would make me feel, you know?...Wow, I managed to get here today...As children have at school, the green, yellow, and red stars...Maybe I’m being childish...but for those who are at this stage...Positive reinforcement is needed....

BCS11

Similar to BCS11, the MHPs participating in the study also recommended the integration of data visualization dashboards into the program. Professionals considered the inclusion of a “simple dashboard providing digested information about specific scores, or cut-offs being exceeded or any tasks or questionnaires pending” (MHP7) would facilitate the handling of the program and BCSs’ treatment progress follow-up. The inclusion of gamification principles was also mentioned as interesting by a few MHPs, owing to its potential of promoting engagement to treatment in BCSs.

The use of unfamiliar terminology (eg, users/*utilizadores*, treatment modules/*módulos de tratamento,* and worksheets/*Fichas de trabalho*) and a perceived lack of integration between some sections of the platform was also a matter of concern. Various participants struggled to grasp iNNOVBC’s affordances because the designations used were unfamiliar to them. Moreover, various participants verbalized difficulties in learning and remembering how to navigate from the user hub or the treatment modules section to the conversations section and vice versa. During this process, errors were recurrent, and many participants opted for a trial and error or a *go to landing page* strategy to complete the proposed tasks. These difficulties hindered not only participants’ effectiveness and efficiency but also their satisfaction with the program:

The terms used weren’t completely obvious...It was hard for me to understand how to find the patient, how to consult the things that she had performed, the tasks I had assigned to her...It confused me because those were not the terms I use in my practice...I believe something closer to what I use daily, closer to the platforms we have there [at the hospital] would be easier...like a list of patients...clinical file...prescriptions...results....

MHP12

Imagine I am reading something, and I am not understanding it well or I want to tell what is happening to me to the therapist...Just the fact that I must go to another page and look for it [conversations section] creates a huge mess because I do not know where to go and when I get there I do not remember anymore where I was at....

BCS7

...I would have to have a paper beside me to write down the doubts that are arising, or I would have to use two screens...Minimizing one to get to the other...It would make some sense, yes, to have it [the chat] always available.

BCS2

As mentioned by BCS2, various participants considered that some features, such as the conversation’s menu, should be always on display. Survivors considered it to be important to express doubts, concerns, and emotions while reading the treatment modules or performing the exercises. Therapists expressed the need to easily provide feedback to their clients and consult communication logs while executing other tasks. In addition, some participants suggested that a customizable toolbar should be made permanently available to facilitate the use of the platform.

The importance of being able to customize the program and use it in a flexible way was reiterated by various participants. The possibility of selecting the treatment modules to work with, how the information is conveyed (eg, audio, video, and text), and between alternative versions of the same material (eg, male and female relaxation audios) was considered important to make the program more inclusive and engaging:

Audios and videos...If the person doesn’t want to read or has a lot of difficulties in understanding, that could help...and, the way I see it, this shouldn’t be an obligation, but an app that is available to help me and to be used according to my needs. For example, the relaxation...I am working on my sleep isn’t it, controlling my sleep and then I could access the other menus like...Now I am going to relax for a while, and then have the exercise 1, 2 and 3 and...ok, today I’m going to use this one....

BCS8

Maybe I like having more control...But I believe it’s important to me to be able to decide which module a specific patient should be working with in a particular week...Depending on what I think that makes more sense to the person at that specific time...or to be the client herself deciding what makes more sense...I believe that for this to be accepted in the future, professionals must have the possibility of adding information or questionnaires themselves. I think that’s important, to make them feel this is a valuable tool and not to reject it.

MHP13

Similar to MHP13, various participants mentioned the intention to use the program as an *à la cart* platform or self-care toolbox, where different content and strategies could be prescribed simultaneously or used as needed. This was also clear from the participants’ behavior during the usability tests, where sometimes errors were committed because participants assumed that modules (eg, the anxiety, sleep, and relaxation modules) could be used interchangeably and not sequentially. This discrepancy between the program’s original concept and how participants appropriated the program or intended to use it was emphasized during the field trials, impacting participants’ perception of iNNOVBC’s feasibility.

### Feasibility

Many participants reported that integrating iNNOVBC into their routine, as prescribed, would be feasible but challenging. MHPs worried about the implementation of the program at their workplaces owing to interoperability as well as practical and attitudinal limitations, whereas BCSs anticipated difficulties in “finding the time to fit the program into the day-to-day life” (BCS1):

Considering the hustle of professional life, I believe it wouldn’t be easy for me to comply with everything that is asked...to enter the site and make daily registrations because I would have to be available to complete several steps and to pay close attention to it. Nowadays, people want simple, fast, and short tasks...and as is, I am not sure I would be able to do it straightforwardly.

BCS6

It is important to me to have some flexibility...I don’t want to make this another chore that I have to do. I think it would make me even more stressed because I would be worried about complying...I don’t want it to be another obligation.

BCS3

As mentioned by BCS3 and BCS6, various interviewees underlined the role that flexibility, accessibility, and ease of use could play in iNNOVBC’s uptake. During the field trials, various participants used iNNOVBC on their smartphones, owing to its portability and usability. A participant who was retired justified that she did not “use computers in a long time, being more acquainted with mobile devices” (BCS4). Another participant who was on sick leave mentioned not using computers regularly, because she did not usually carry it with her anymore and some “movements, like pulling the plug or pressing the mouse buttons, still hurt due to hormonal treatment” (BCS9). Another working participant revealed being receptive to use the web version of the program but showed some resistance in spending additional hours in front of the computer while at home (BCS10). However, as iNNOVBC runs on a responsive web-based platform, some materials did not perfectly adapt to their mobile devices and some exercises such as the relaxation audios were interrupted by notifications, thereby compromising the delivery of the intervention. Thus, some participants reported it would “be easier to have it in app format” (BCS11), suggesting that this is a more convenient and accessible format for BCSs.

Another important aspect discussed by BCSs as possibly facilitating adherence to the program concerned having a direct communication channel with psychologists:

Having a psychologist on the other side of the screen is particularly important...Sometimes you need to listen to someone assertive to be able to keep going...If this were like an online helpline like SNS24 [a telephone and web-based service of the Portuguese National Health Service that provides support to citizens when they need advice with acute, nonemergent health complaints] but for psychological issues, I would use it often. If I saw it was reliable and that it helped me, I believe I would use it several times, because I missed that support.

BCS7

Having timely feedback on what to expect or about what is considered normal and not having to wait for the next appointment could be very important to better manage the emotional impact of cancer, making me interested in using this.

BCS10

The possibility of posing doubts; discussing difficulties associated with the treatment, follow-up, and discharge processes; and receiving professional feedback from psychologists was valued by most BCSs. Interestingly, survivors seemed to conceptualize iNNOVBC not necessarily as a structured psychotherapeutic approach but as an on-demand tangible supportive tool that could provide them with access to tailored content and psychological support timely delivered according to the specific moment of the survivorship trajectory they were at. According to participants, both information and supportive care needs change significantly along the survivorship continuum and having access to such a tool could secure them that they were “still being taken care of” (BCS1) during the follow-up stage.

However, to fulfill its full supportive role, besides performing some simplifications to the program, most participants considered that some sort of training should be provided to both MHPs and BCSs. Participants considered that having access to training material in graphical, written, or video format would facilitate the use of the program. In addition, some MHPs mentioned that considering the novelty of the programs’ approach, the provision of training sessions and on-job training would be advisable not only to present the platform but also to introduce therapists to the program’s rationale and components:

It is important to know more about the program, how it was created and that it really works. It would provide us with more confidence in using the program if we knew we would not be wasting our clients’ time and money.

MHP9

I believe training should be an extended version of what we have done here today [usability test], with practical situations and instructions, so we can clarify our doubts...like a workshop session...and then having someone there to help us during the first weeks....

MHP12

The positive impact of having dedicated professionals, conceivably digital navigators, supporting the implementation of the program was also discussed by BCSs. Some participants considered it would be important to have an appointed professional to introduce them to iNNOVBC’s content, structure, and features, at their cancer centers. Such support could help them in overcoming usability issues and attitudinal barriers toward the program and, ultimately, facilitating adoption:

After being admitted or having a first appointment with the doctor, he could say “now you are going to meet a colleague of mine, that will show you how to use a tool that can help you deal with your situation” and then the designated colleague, that has to be an appealing person, possibly a psychologist to better know how to convey the information to the person, would explain the purpose of the program, show how the program works and even trial it with the patient...in the beginning, they might think “this is boring”, but If people are properly introduced [to the program], later, when they are feeling more anxious, they might remember “Ah, I have that app that can help me 24/7” and they would value the support that is provided in here.

BCS8

iNNOVBC was regarded as part of a comprehensive portfolio of services to be provided by cancer centers to BCSs, to which they would have to be informed about, introduced to, and properly referred to, to be able to use it at its full potential. Similar to BCS8, some MHPs thought that oncologists could play a significant role in facilitating iNNOVBC dissemination and uptake in cancer settings. As physicians “are the ones orchestrating treatment” (MHP5), they were viewed by participants as important gatekeepers of programs such as iNNOVBC. However, some MHPs mentioned that oncologists could not necessarily be “receptive to prescribe it, due to their biomedical approach...and lack of involvement in the delivery of psychosocial interventions” (MHP2). Thus, for iNNOVBC to become part of survivors’ “adjuvant supportive care” (MHP1) and for it to be properly integrated into clinical settings, some MHPs considered that training should be extended to other professional groups, such as nurses, physicians, and managers working within oncology. To be successfully implemented, iNNOVBC would have to be recognized as a valuable service to be provided by the several actors playing in the cancer setting.

## Discussion

### Principal Findings

In this study, iNNOVBC—a guided, internet-delivered, individually tailored, ACT-influenced CBT intervention aiming at treating mild to moderate anxiety and depression as well as improving fatigue, insomnia, sexual dysfunction, and HRQoL in BCSs—was developed with a user-centered design approach [37] and explored concerning its usefulness, usability, and feasibility.

Overall, participants considered iNNOVBC highly useful, with most interviewees reporting on the pertinence of its scope, digital format, content, and features. Consistent with the literature [63], participants reported on the high prevalence of physical, emotional, practical, and information unmet care needs experienced by BCSs and considered that iNNOVBC could help bridge the supportive care gap experienced by BCSs across the survivorship trajectory. Similar to previous research [64-66], survivors valued having access to ubiquitous evidence-based self-care information that was organized in accordance with the cancer continuum, considering that it could help them better manage and cope with their condition and problems.

Another important aspect contributing to participants’ perception of the usefulness of iNNOVBC concerned the multifeatured and integrated nature of the program. Participants appreciated the fact that iNNOVBC combined psychoeducation, communication, documentation, and automatized scheduling and notification features, allowing a more efficient and comprehensive assessment and follow-up of BCSs. Furthermore, the possibility of MHPs and BCSs to communicate synchronously and asynchronously (eg, via chat, email, and videoconference) through the platform, as well as in accordance with different degrees of personal exposure, was valued by various participants. Some participants mentioned that using chat or email could facilitate self-disclosure in BCSs and promote the timely discussion of sensitive topics often avoided, thus having a positive impact on the established therapeutic alliance and process. Nevertheless, few studies have addressed chat-based internet interventions targeting cancer survivors. Previous research has focused on chat groups for patients with prostate cancer [67] or adolescents treated for cancer and reported mixed results. Thus, it is necessary to conduct further research on one-to-one chat-based programs to evaluate the role chat sessions could have in survivors’ treatment progress and engagement in digital supportive care.

Despite participants’ perceived usefulness of iNNOVBC, both BCSs and MHPs identified aspects hindering their experience while using the program and changes to be performed to achieve better effectiveness, efficiency, and satisfaction with iNNOVBC. These included refining the program’s aesthetics by using a minimalist and recognizable design; improving interaction design by making the navigation within the program more consistent and constrained; decreasing the cognitive overload experienced by participants by using terminology tailored to each context of use and balancing the amount of information displayed; increasing the feedback provided to users, so that they are continuously informed about the impact of their work within the program; and diversifying the media used for intervention delivery by developing smartphone and tablet versions of the program. These findings echo many of the limitations and development requirements gathered in previous research aimed at developing digital programs for supporting cancer survivors [64,65,68-73].

In particular, BCSs and MHPs participating in this study requested the simplification of navigation paths, suggesting that the use of an opening dashboard would facilitate the understanding and handling of iNNOVBC. Adding labels and preview options to the most used functionalities was also considered important to increase the discoverability of the program. Likewise, the use of a more dynamic color scheme and greater use of icons, images, and shortcuts were appointed as necessary to facilitate the recognition of the program’s affordances and facilitate its use. Previous research has yielded comparable results [64,65,69-71], suggesting that single-page websites or apps that enable properly labeled interactions and make use of different color depths, familiar icons, and images are more usable and acceptable to cancer survivors.

Moreover, participants underlined the importance of balancing and diversifying the information that is displayed at each given time, as well as simplifying data entry tools. According to participants, information should be grouped more concisely (eg, short modules addressing a single theme), delivered using various media (eg, web, smartphone, and tablet), and displayed in different formats (eg, text, audio, and video) and versions (eg, female and male audio clips) and should allow entering data using plain textboxes instead of tables to make the program more inclusive and easier to use. Complementarily, the integration of data visualization dashboards providing digested information on completed or to-be-completed tasks and questionnaires, its scores, and cutoff points being exceeded or achieved was appointed by interviewees as relevant. Participants anticipated that such a strategy could facilitate the handling of the program and the assessment of BCSs’ treatment progress, promoting their engagement with iNNOVBC. Previous research corroborate these findings [71-73]. In a previous study by Igelström et al [71], the importance of delivering content in different formats and adding a graphical display of self-reports was emphasized by participant survivors. In another study by Wagner et al [72], BCSs stressed the importance of developing a *my progress page* to display didactic content and tools that had been completed and chart anxiety scores to facilitate tracking of progress. Nevertheless, and according to Kuijpers et al [74], although professionals might be primarily interested in dashboards indicating a worsening of symptoms to help patients reduce symptom burden, survivors seem to be interested in monitoring changes in their symptom experience and functional health, preferring to see both worsened and improved scores depicted in such visualizations. These results underline the importance of tailoring DMH programs to the profile and unique preferences of each user to better address their concerns and needs.

The option to tailor iNNOVBC along the cancer continuum was a salient development requirement identified during this study. Being conceived as an individually tailored program, iNNOVBC permits some degree of tailoring, namely, in what concerns the treatment modules. However, participants considered further layout and content customization should be allowed so that the program could adapt to the idiosyncrasies of each of the survivorship trajectory stages. Participants considered that comprehensive support should be provided along this continuum, not compartmentalizing survivors’ needs but shaping and transforming the program according to its evolution. Similar to a rhapsody, iNNOVBC should adopt “an episodic yet integrated, free-flowing structure, featuring a range of highly contrasted moods, colour, and tonality” [75], to be used freely and flexibly as needed. This finding aligns with previous research [66,72,76,77] and emphasizes the importance of building flexibility into digital programs targeting cancer survivors, not only in terms of tailoring but also in terms of frequency and timing of use, to ensure its uptake by target users.

However, such an understanding of the program contrasts with its original concept and theoretical grounding. Although participant survivors mentioned the intention to use the program as an *à la cart* platform or self-care toolbox, where different content and strategies could be prescribed simultaneously or used as needed, and not in a prescriptive manner, evidence suggests that the implementation of structured approaches, theoretically grounded and validated to specific contexts of use, best serves survivors [13,14]. This discrepancy adds to the technical, usability, funding, attitudinal, and training limitations identified by participants and the literature [51] as potentially hindering successful implementation of iNNOVBC and underlines the need to further assess and refine it in clinical contexts before scaling up the program. Thus, iNNOVBC will soon be pilot-tested in cancer settings [78] not only to assess its preliminary efficacy but also to further assess its feasibility and gather requirements for the design of a patient-centric service that fits into BCSs’ lives, professionals’ evidence-based practices, and cancer centers’ workflows. After piloting and further refining iNNOVBC, the program will be tested for its efficacy and cost-effectiveness using a multicenter, randomized, waiting list, controlled design [78]. The results from this parent study will determine whether iNNOVBC should be transferred to routine care.

### Strengths and Limitations

This study presents various strengths and limitations. Strengths of this study include the adoption of a user-centered design approach combining mixed methods (eg, surveys, in-depth interviews, usability tests, and field trials) that were used to explore the usefulness, usability, and preliminary feasibility of iNNOVBC in a comprehensive manner. Furthermore, the study was conducted by an interdisciplinary team and involved iNNOVBC’s primary and secondary end users, that is, BCSs and MHPs, benefiting from complementary input by these stakeholders. In addition, participants have been purposefully sampled for diversity in age, academic degree, and digital technology proficiency. Limitations include a small sample size and minimal diversity in participants’ educational level and experience in using DMH programs. Moreover, participant BCSs were younger in the current sample than the general Portuguese BCS population [79], and participant survivors were not screened for mild to moderate anxiety or depression. This aspect may have prevented the identification of usability issues associated with older age or psychological morbidity, thereby limiting the overall generalizability of our results. Nevertheless, there is some consensus that usability tests may include a minimum of 5 participants per iteration and that approximately 80% of usability issues are discovered with as few as 4-6 participants [80]. Furthermore, many of the results obtained in this study align with previous research, supporting its ecological validity. Future research [78], aiming at pilot-testing and further assessing iNNOVBC’s feasibility, efficacy, and cost-effectiveness, should include older and less technically adept participants to complement the findings of this study.

### Implications for the Design and Implementation of DMH Programs in Cancer Settings

The results of this study hold important implications for the further development and implementation of programs such as iNNOVBC. First, DMH programs targeting cancer survivors could benefit from the early involvement of its primary, secondary, and tertiary end users in the development process. By involving survivors, health care professionals, and managers in codevelopment activities (eg, surveys, in-depth interviews or focus groups, usability tests, and field trials), interventions could be designed to address stakeholders’ real needs and development could better align with their practices and contexts, thereby increasing the odds of successful implementation. Second, involving interdisciplinary teams in the development process is key to ensure that comprehensive solutions are developed and design caveats are timely anticipated, identified, and refined, thus not compromising the usefulness, usability, and feasibility of such programs. Third, DMH interventions must be conceived as supportive point-of-need services capable of adjuvating and extending cancer care delivery. Programs must adopt a flexible yet integrated structure capable of being continuously tailored to end users’ changing needs, evolving along the cancer continuum. In this context, transdiagnostic programs might be particularly useful in fulfilling this requirement. Finally, implementation research must be conducted to determine the effectiveness of developed programs and identify service delivery bottlenecks (eg, lack of training) to which fast-track solutions (eg, digital navigators) must be developed and tested.

### Conclusions

This study explored the usefulness, usability, and preliminary feasibility of iNNOVBC, and its results suggest that DMH programs, such as iNNOVBC, are considered useful by both BCSs and MHPs, thus configuring a promising point-of-need solution to bridge the supportive care gap experienced by BCSs across the survivorship trajectory. However, to fulfill its full supportive role, such programs must be comprehensive, highly usable, and tailorable and adopt a flexible yet integrated structure capable of evolving in accordance with survivors’ changing needs along the cancer continuum.

## Appendix

Multimedia Appendix 1 Study intervention components.

Multimedia Appendix 2 Breast cancer survivors’ and mental health professionals’ interview scripts.

Multimedia Appendix 3 Breast cancer survivors’ and mental health professionals’ iNNOV Breast Cancer usability test protocol and observation tables.

Multimedia Appendix 4 Breast cancer survivors’ and mental health professionals’ debriefing interview scripts.

Multimedia Appendix 5 Field trial protocol.

Multimedia Appendix 6 Usefulness and feasibility questionnaire.

Multimedia Appendix 7 Identified usability issues.

## Abbreviations

ACT: acceptance and commitment therapy
BCS: breast cancer survivor
CBT: cognitive behavioral therapy
DMH: digital mental health
HRQoL: health-related quality of life
iNNOVBC: iNNOV Breast Cancer
MHP: mental health professional
