# Supplementary material for: Development and Evaluation of the Usefulness, Usability, and Feasibility of iNNOV Breast Cancer: Mixed Methods Study
Source: JMIR Cancer. 2022 Feb 15;8(1):e33550. doi: 10.2196/33550 (PMC8889471; doi:10.2196/33550)
Supplement: Multimedia Appendix 1 [file cancer_v8i1e33550_app1.docx]

**Multimedia Appendix 1: Study intervention components**

|  |  |  |
| --- | --- | --- |
| **Module** | **Core Processes and Intervention Techniques** | **Content** |
| **About this treatment** (Week 0) |  | - General introduction to iNNOVBC aims, theoretical framework, structure, and format. Introduction to iTerapi’s functionalities. |
| **Living with breast cancer and beyond**  (Week 1) | - Psychoeducation - Contextual Functional Analysis - Values | - Information about breast cancer; the survivorship trajectory; and the common late side effects and psychological comorbidities associated to breast cancer. - Introduction to Cognitive Behaviour Therapy (CBT) and Acceptance and Commitment Therapy (ACT) rationale: the relationship between cognition, emotion, and behaviour; the ABC model; values as chosen life directions; and how to set treatment goals. - *Activities:* Identifying and hierarchizing values; setting treatment goals; Telling my story. |
| **Depression**  (Week 2) | - Psychoeducation - Acceptance - Cognitive Defusion | - Information about Unhappiness and depression; depression aetiology, signs, and symptoms; feelings, behaviours, and thoughts in depression; and feeling depressed after breast cancer. - Introduction to experiential avoidance and acceptance concepts. - *Activities:* Identifying sources of discomfort and the actions taken to ease the discomfort; Naming and observing inner experiences. |
| **Behavioural Activation: Part I** (Week 3; Optional) | - Psychoeducation - Behavioural activation - Committed Action | - Introduction to behavioural activation rationale. - *Activities:* Activity Scheduling/ Activity Plan; My positive activities; My negative activities; Increasing the probability of executing positive activities; Evaluating and redesigning your Activity Plan. |
| **Behavioural Activation – Part II** (Week 4; Optional) | - Psychoeducation - Behavioural activation - Committed Action | - Introduction to negative activities and strategies to manage negative activities. - *Activities:* Dividing negative activities into smaller activities; Examining previous week Activity Plan; Designing a reward list; Evaluating and redesigning your Activity Plan to integrate previously identified rewards. |
| **Anxiety** (Week 3 or 5) | - Psychoeducation - Cognitive Defusion - Exposure | - Information about anxiety, worries and fear of recurrence and strategies to manage anxiety. - Activities: Anxiety Ladder; Planning a time to worry and feel anxious (includes worries diary); Exposure to an object, situation, or thought. |
| **Relaxation** (Week 4 or 6) | - Psychoeducation - Relaxation techniques | - Introduction to relaxation rational and techniques. - *Activities:* Progressive muscle relaxation; Applied relaxation - diaphragmatic breathing, using a relaxation word, faster relaxation, relaxing in anxious situations. |
| **Sleep problems**  (Week 5 to 9; Optional) | - Psychoeducation - Sleep management techniques | - Information about sleep, sleeping problems and sleep management techniques. - *Activities:* Mapping your altering sleep factors; choosing and implementing your sleep management techniques: a) Sleep hygiene, b) Relaxation; c) Stimulus control; d) Sleep restriction. |
| **Fatigue**  (Week 5-9; Optional) | - Psychoeducation - Energy conservation techniques | - Information about fatigue and strategies to cope with it. - *Activities:* Fatigue diary; 6P energy conservation techniques; Identifying fatigue-promoting occupations and identifying energy conservation strategies to adapt and deal with these occupations; prioritizing my activities and planning my day. |
| **Interpersonal relationships, Sex, and Intimacy** (Week 5-9; Optional) | - Psychoeducation - Problem solving - Sensate focusing | - Interpersonal relationships, sex, and intimacy difficulties in survivorship; - *Activities:* Practical problem solving; sensate focusing. |
| **Conclusion: Key points summary and Planning for the future**  (Week 5 or 10) | - Psychoeducation - Relapse prevention | - Summary of the treatment program; Therapy goals reassessment; Overview and assessment of the learned techniques; Preparing for the unexpected; Setbacks and relapse.; Situations where setbacks can occur; Planning for maintenance and relapse prevention. |
