# Supplementary material for: Development and Evaluation of the Usefulness, Usability, and Feasibility of iNNOV Breast Cancer: Mixed Methods Study
Source: JMIR Cancer. 2022 Feb 15;8(1):e33550. doi: 10.2196/33550 (PMC8889471; doi:10.2196/33550)
Supplement: Multimedia Appendix 2 [file cancer_v8i1e33550_app2.docx]

**Multimedia Appendix 2: BCS and MHP interview scripts**

**Breast cancer survivors’ interview script** (in Portuguese followed by a translation to English)

Warm-up:

Pode por favor descrever-me o seu dia-a-dia? (*Can you please describe a typical day?*)

Sente dificuldade na realização de alguma atividade no seu dia-a-dia? (*Do you have any difficulty performing your daily activities?*)

Se sim. Alguma dessas dificuldades foi causada pelo cancro da mama ou seus tratamentos? (*Have any of these difficulties resulted or been aggravated by the diagnosis of breast cancer?)*

Se não. Em que medida é que a sua rotina se alterou com o diagnóstico de cancro da mama? (If not. *How has your routine changed with the diagnosis of breast cancer?*)

Cancer history:

Pode dar-me mais detalhes sobre a doença que teve? (*Can you tell me a little about the disease you had?*)

Pode falar-me um pouco sobre o seu tratamento? (*Can you tell me a little about your treatment?*)

O que é que foi mais difícil gerir nessa fase? (*What was harder to manage at this stage?)*

Survivorship:

Como se sentiu quando concluiu o tratamento primário (cirurgia/QT ou RT, dependendo do tratamento prescrito)? (*How did you feel when you completed primary treatment (Surgery, CT or RT)?*)

Quais foram as maiores dificuldades que sentiu nessa altura? (*What were the biggest difficulties you felt at that time?*)

Pode por favor descrever os seus pensamentos nessa fase? (*Can you please describe your thoughts during that period?*)

Unmet needs/Personal self-care strategies:

Usou alguma estratégia pessoal para lidar com essas dificuldades? (*What personal strategies did you use to deal with these difficulties?*)

Como descreveria o apoio que recebeu por parte de terceiros, para lidar com essas dificuldades (equipa médica, família, amigos, etc.)? (*How would you describe the support you received from others to deal with these difficulties (clinical staff, family, friends, etc.)*?

Em que medida é que esse apoio a ajudou a ultrapassar essas dificuldades? (*To what extent has this support helped you overcome those difficulties?*)

Quais os principais desafios com que se deparou, do ponto de vista emocional? (*What are the main challenges you faced emotionally?*)

Psychiatric antecedents:

Alguma vez foi acompanhada em consultas de psicologia ou psiquiatria? (*Have you ever been followed by a psychologist or psychiatrist?*)

Se sim. O que a fez procurar apoio psicológico? (If yes. *What made you seek psychological support?*)

Se sim. Como descreveria essa experiência? (If yes. *How would you describe this experience?*)

Se sim. Teve alguma dificuldade em aceder a esses cuidados? (If yes. *Did you experience any difficulties accessing that support?*)

Se não. Qual acha que teria sido o impacto de ter sido acompanhada por um psicólogo ou psiquiatra? (If not. *What would have been the impact of being followed by a psychologist or psychiatrist?*

Em que fase é que acha que este tipo de apoio é mais necessário? (*At what stage is this type of support most needed?*)

Attitudes towards internet interventions:

Alguma vez recebeu ou ponderou procurar apoio psicológico online? (*Have you ever received or considered seeking online psychological support?*)

Se sim, pode falar-me um pouco mais sobre isso? (*Can you elaborate on that?*)

Que vantagens/desvantagens associa a esse tipo de apoio? (*What advantages/disadvantages do you associate with this type of support?*)

Que características deveria ter um serviço deste género? (*What characteristics should such a service have?*)

Se a unidade de saúde onde se encontra a ser seguida disponibilizasse esse tipo de apoio ponderaria utilizá-lo? *(If the health unit you are followed at provided such support, would you consider using it?)*

O que é que influenciaria a sua decisão de utilizar ou não uma solução deste género? (*What would influence your decision whether to use or not such solution?*)

Digital technology use/proficiency:

Como caracteriza a sua experiência de utilização de tecnologias de informação e comunicação (e.g. PC, smartphone, internet, etc.)? (*How do you characterize your experience using information and communication technologies (eg PC, smartphone, internet, etc.)?*)

Já usou alguma tecnologia para manter-se saudável, controlar uma doença ou para se tratar? (*Have you ever used technology devices or online resources to be healthy, control a disease or to treat yourself?*)

“Obrigada X. Se estiver de acordo, passaremos agora ao teste de usabilidade.” *(Thank you. If you agree, we will now move on to usability testing.)*

**Mental Health Professionals’ interview script** (in Portuguese followed by a translation to English)

Warm-up/professional background:

Pode por favor descrever-me um dia normal de trabalho? (*Can you please describe a typical working day?*)

Que tipo de clientes/doentes costuma acompanhar? (*What type of clients / patients do you usually work with?*)

Com que frequência consulta doentes oncológicos? (*How often do you see cancer patients?*)

Se frequentemente. Quais as problemáticas mais frequentemente reportadas por este tipo de doentes? (If often. *What are the problems most frequently reported by these patients?*)

Se frequentemente. Recorda-se de alguma estratégia de autocuidado que algum dos seus doentes tenha referido utilizar para lidar com estas dificuldades? (If often. *Do you recall any self-care strategy that any of your patients reported using to deal with these difficulties?*)

Se frequentemente. Considerando esta população, qual a fase da doença/processo de tratamento em que o acompanhamento psicológico lhe parece mais crítico? (If often. *What is the stage of the disease/treatment process in which psychological counseling is most critical in this population?*)

Considerando o seu contexto de trabalho, como caracterizaria o acesso a cuidados de saúde mental por parte dos clientes/doentes? (*Given your work context, how would you characterize clients / patients' access to mental health care?*)

Knowledge and Use of internet interventions:

Alguma vez utilizou tecnologias digitais, tais como a internet ou aplicações, para prestar apoio psicológico aos seus clientes/doentes? (*Have you ever used digital technologies, such as the internet, to provide psychological support to your clients / patients?*)

Se sim. Pode falar-me um pouco sobre essa experiência (tipo de tecnologia usada/em que contexto/objetivo da intervenção/pedido do cliente ou sugestão do próprio/indicação/duração da relação terapêutica/em exclusividade ou complementaridade/aceitabilidade por parte cliente/doente)? (If so. *Can you tell me a little about this experience (type of technology used / in what context / purpose of intervention / client request or own suggestion / indication / duration of therapeutic relationship / in exclusivity or blended / acceptability by client / patient?*)

Se não. Pondera utilizar este tipo de intervenções no futuro? (*If no. Do you plan to use such interventions in the future?*)

Se sim, em que circunstâncias (motivação/indicações/exclusividade vs complementaridade)? (*If so. Under what circumstances (motivation / indications / exclusivity vs complementarity)? )*

Se não. O que o/a demove de utilizar esse tipo de intervenções? (If no. *What prevents you from using these interventions?*)

O que entende por/Como descreveria as intervenções psicológicas mediadas por tecnologias digitais? (*What do you understand by / How would you describe this type of interventions?*)

Attitudes towards internet interventions:

Qual a sua perspetiva acerca deste tipo de intervenções? (*What is your perspective on such interventions?*)

Quais são para si as principais vantagens associadas a esse tipo de intervenções (acessibilidade/conveniência/económicas/redução estigma/privacidade e anonimato/equidade/empowerment clientes/personalização cuidados/evidência científica)? (*What are for you the main advantages associated with such interventions (accessibility / convenience / economic / stigma reduction / privacy and anonymity / equity / customer empowerment / care personalization / scientific evidence)?*)

E limitações (iliteracia informática/ética/atitudes negativas/aspetos culturais/segurança sistemas/dificuldades implementação/políticas/económicas)? (*What about limitations (computer illiteracy / ethics / negative attitudes / cultural aspects / security systems / implementation / political / economic difficulties)?*)

Pode dar-me exemplos de situações em que tenha experienciado essas dificuldades ou mais-valias? (*Can you give me examples of situations in which you have experienced these difficulties or advantages?*)

Como qualificaria a evidência científica relativa à eficácia e efetividade deste tipo de intervenções? (*How would you qualify the existing scientific evidence regarding efficacy and effectiveness of such interventions?*)

Considera que a utilização de tecnologias digitais para prestação de cuidados de saúde mental constituem um risco para os profissionais da área? Se sim, em que medida? (*Do you consider that the use of digital technologies for mental health care is a risk for practitioners? If so, to what extent?*)

O que pensa sobre a possibilidade de conduzir um processo de avaliação psicológica à distância? (*What do you think about the possibility of conducting a psychological assessment process at a distance?*)

Como caracterizaria este tipo de intervenções no que se refere à segurança e confidencialidade? (*How would you characterize such interventions regarding security and confidentiality?*)

De acordo com a sua perspetiva, este tipo de intervenções levanta algum desafio ético? Se sim, quais? (*According to your perspective, do this type of interventions raise any ethical issues?)*

O que pensa sobre a possibilidade de acompanhar clientes/doentes que mantenham a sua identidade anónima? (*What do you think about consulting clients / patients who maintain their identity anonymous?*)

Qual prevê que seja o impacto deste tipo de intervenções ao nível do processo terapêutico? (*What do you expect to be the impact of such interventions on the therapeutic process?*)

Antecipa algum tipo de dificuldades no que se refere à adaptação dos protocolos terapêuticos que utiliza, ao contexto digital? Se sim, quais? (*Do you anticipate any difficulties in adapting the therapeutic protocols you use to the digital context? If so, which ones?)*

Qual prevê que seja o impacto deste tipo de intervenções nos clientes/doentes no que diz respeito ao seu empoderamento e autocuidado? (*How would this type of interventions impact patients’ empowerment and self-care?*)

Considera que este tipo de intervenções poderão comprometer o controlo sobre o processo terapêutico, por parte dos profissionais de saúde? Se sim, de que forma? (*Do you think this type of intervention could compromise the control of the therapeutic process by health professionals? if so, in what sense?*)

Antecipa algum tipo de dificuldade na gestão de situações de crise neste contexto? (*Do you anticipate any difficulties in managing crisis situations in this context?)*

Como caracteriza o estabelecimento da aliança terapêutica neste contexto?/ Como pensa que se estabeleceria a aliança terapêutica neste contexto? (*How do you characterize the establishment of the therapeutic alliance in this context? / How do you think the therapeutic alliance would be established in this context?*)

Apesar das novas tecnologias estarem amplamente difundidas na nossa sociedade, a implementação deste tipo de intervenções, no SNS, prática clínica privada, etc. é limitada. De acordo com a sua perspetiva, que aspetos poderão estar a atrasar esta implementação? (*Although new technologies are widespread in our society, the implementation of such interventions in the NHS, private clinical practice, etc. is limited. What do you think may be delaying this implementation?*)

Em que medida é que a utilização deste tipo de intervenções por parte de outros colegas influenciaria a sua adoção das mesmas? (*To what extent would the use of internet interventions by other colleagues influence your adoption of such interventions?*)

Digital technology use/proficiency:

Como caracteriza a sua experiência de utilização de tecnologias de informação e comunicação (e.g. PC, smartphone, internet, etc.)? (*How do you characterize your experience using information and communication technologies (eg PC, smartphone, internet, etc.)?*)

Alguma vez teve formação acerca de intervenções psicológicas mediadas por tecnologia digital? (*Have you ever received training in internet interventions?)*

“Obrigada X. Se estiver de acordo, passaremos agora ao teste de usabilidade.” *(Thank you. If you agree, we will now move on to usability testing.)*
