# Supplementary material for: Development and Evaluation of the Usefulness, Usability, and Feasibility of iNNOV Breast Cancer: Mixed Methods Study
Source: JMIR Cancer. 2022 Feb 15;8(1):e33550. doi: 10.2196/33550 (PMC8889471; doi:10.2196/33550)
Supplement: Multimedia Appendix 3 [file cancer_v8i1e33550_app3.docx]

**Multimedia Appendix 3: BCS and MHP iNNOVBC usability test protocol and observation tables**

**Breast Cancer Survivors’ usability test protocol and observation table**

| **Participant #:** | **Location:** | **Date:** | **Starting time:** | **End time:** |
| --- | --- | --- | --- | --- |
|  |  |  |  |  |

| **Group** | **Task nº:** | **Script** | **Description** | **P/F** | **A** | **Notes** |
| --- | --- | --- | --- | --- | --- | --- |
| A: login | Task 1 | *“Aceda por favor à plataforma, utilizando as credenciais que lhe foram fornecidas.”*  *"Please access the platform, using the provided credentials."* | Log in to INNOVBC platform with given credentials |  |  |  |
| B:Notifications | Task 2 | *“Verifique se há alguma ação a realizar antes de prosseguir e aja de acordo.”*  *"Check if there is any action to take before proceeding."* | Check notifications |  |  |  |
|  | Task 3 |  | Read Therapist’s message with instruction to the following task. |  |  |  |
| C: Treatment content management | Task 4 | Written on the platform: *“Leia acerca de como realizar o relaxamento muscular profundo que é uma técnica de relaxamento para gerir a ansiedade”.*  *"Read about how to perform deep muscle relaxation which is a technique for managing anxiety"* | Access treatment modules |  |  |  |
|  | Task 5 |  | Access the relaxation module |  |  |  |
|  | Task 6 |  | Open deep muscle relaxation page and expand the “how to practice” text. |  |  |  |
|  | Task 7 | *“Ouça a gravação da sessão de relaxamento disponível nesta página”.*  *"Listen to the recording of the relaxation session available on this page".* | Play the recorded relaxation session |  |  |  |
|  | Task 8 | *“Grave esta sessão de relaxamento no desktop/ambiente de trabalho deste computador”.*  *"Save the relaxation session on this computer's desktop."* | Download the relaxation session |  |  |  |
|  | Task 9 | *“Imprima o conteúdo da página”.*  *"Print the page content".* | Print the current page |  |  |  |
| D: Worksheets completion | Task 10 | *“Pedia-lhe agora que localizasse o exercício relativo à ansiedade, que se encontra na secção 8 desse capítulo”.*  *"Please find the exercise on anxiety, which is on page 8."* | Return to the modules |  |  |  |
|  | Task 11 |  | Access page 8 of the anxiety module |  |  |  |
|  | Task 12 | *“Introduza agora a palavra “aranhas” no menor nível de ansiedade identificado neste exercício e torne este registo permanente antes de regressar ao princípio”.*  *"Now enter the word" spiders "in the lowest anxiety level identified in this exercise and make this record permanent before returning to the beginning".* | Complete and save anxiety ladder exercise. |  |  |  |
|  | Task 13 |  | Return to homepage |  |  |  |
|  | Task 14 | *“Gostaria agora que completasse o diário de sono disponível no capítulo sono desta plataforma” (apenas coluna 1).*  *"Please complete the sleep diary available on this platform" (column 1 only).* | Go to the sleep module and search for the exercise. |  |  |  |
| E: Communicating with therapists | Task 15 | *“Envie por favor um e-mail ao seu psicoterapeuta a dizer que está disponível para falar agora com ele”.*  *"Please send an email to your psychotherapist saying that you are available to speak with him now."* | Send an e-mail to therapist |  |  |  |
|  | Task 16 | “*Entretanto recebeu uma resposta deste via e-mail a dizer que também está disponível e a pedir-lhe para iniciar a conversa. Diga por favor “Olá” ao seu psicoterapeuta”*  *“In the meantime, he replied you via e-mail saying that he is also available and asking you to start the conversation. Please say "Hello" to your psychotherapist ".* | Open chat and type “hello” to the therapist via chat |  |  |  |
|  | Task 17 | *“Este responde que é preferível falarem face-a-face. Por favor inicie essa conversa”.*  *“He replies that it is preferable to speak face-to-face. Please start this conversation”.* | Activate the video-conference function and call Cristina |  |  |  |
| F: Scheduling tasks | Task 18 | *“O seu psicoterapeuta sugeriu que marcasse um momento para relaxar hoje ao final do dia e que a plataforma a poderá avisar quando chegar essa altura. Programe por favor essa atividade”.*  *“Your psychotherapist suggested that you schedule a time to relax today at the end of the day and that the platform alert you when that time comes. Please schedule this activity”.* | Go to calendar and schedule a new task named relaxation on the xx/yy/zzzz at xx:yy and set an alarm/notification to 15 minutes prior the event |  |  |  |
| **Instructions:**Register the number of**P –** Pass; **F -**Fail; and **A –** Assistances occurrences. A task will only be completed if the condition of the task is true. An error should be counted every time the participant performs an action that does not contribute to task completion.  An assistance is considered every time the participant requests the assistance of the facilitator to perform the task. If the assistance is required because the task was not well explained it should not be considered as assistance in task completion. If the facilitator intervenes because he feels the participant needs help, it should also be accounted for as an assistance. | | | | | | |

**Mental Health Professionals’ usability test protocol and observation table**

| **Participant #:** | **Location:** | **Date:** | **Starting time:** | **End time:** |
| --- | --- | --- | --- | --- |
|  |  |  |  |  |

| **Group** | **Task nº:** | **Script** | **Description** | **P/F** | **A** | **Notes** |
| --- | --- | --- | --- | --- | --- | --- |
| A: login | Task 1 | *“Aceda por favor à plataforma, utilizando as credenciais que lhe foram fornecidas.”*  *"Please access the platform, using the provided credential"* | Log in to INNOVBC platform with given credentials |  |  |  |
| B: Notifications | Task 2 | *“Verifique se há alguma ação a realizar antes de prosseguir e aja de acordo.”*  *"Check if there is any action to take before proceeding and act accordingly."* | Check notifications |  |  |  |
|  | Task 3 |  | Return to homepage or click in ADMIN>Users>Tina Turner or proceed to next task |  |  |  |
| C: Treatment prescription | Task 4 | Written on the platform: *“Verifique quais as componentes da intervenção que foram atribuídas a Tina Turner”.*  *"Check which components of the intervention have been attributed to Tina Turner".* | Click on Tina Turner’s link or Search for Tina Turner’s page to access her profile or go to Users>Tina Turner. |  |  |  |
|  | Task 5 |  | Click in Treatment |  |  |  |
|  | Task 6 | *“Depois de verificar o tratamento que esta doente está a fazer, e baseando-se na avaliação que fez da mesma, decide prescrever-lhe o módulo para tratamento da insónia. Por favor faça essa prescrição de modo a que esse conteúdo fique imediatamente disponível e notificando a doente acerca desta mudança”.*  *“After checking the treatment that this patient is undergoing, and based on your assessment, you decide to prescribe her the insomnia treatment module. Please perform this prescription so that the module is immediately available and notify her about this change.* | Assign the sleep module to Tina Turner selecting “Sono” |  |  |  |
|  | Task 7 |  | Send a notification to Tina Turner |  |  |  |
|  | Task 8 |  | Save the previous procedure |  |  |  |
|  | Task 9 | *Entretanto foi informado(a) que esta doente aceitou participar num Ensaio Clínico a decorrer na instituição. Prescreva pf esse tratamento à doente em questão, estipulando que a data de início é amanhã”.*  *In the meantime, you were informed that this patient agreed to participate in a Clinical Trial taking place at your institution. Prescribe this treatment to the patient, stipulating that the start date is tomorrow”.* | Click on “Protocolo Terapêutico” |  |  |  |
|  | Task 10 |  | Select “Ensaio Clínico INNOVBC” and schedule the onset of the treatment |  |  |  |
|  | Task 11 |  | Save the previous procedure |  |  |  |
| D: Treatment progress assessment | Task 12 | *“A sua doente já iniciou o tratamento e compete-lhe agora enviar-lhe o seu feedback acerca dos trabalhos de casa desta semana, mais especificamente sobre a implementação da técnica de higiene do sono”.*  *"Your patient has already started treatment and it is now up to you to send her feedback about this week's homework, more specifically about the implementation of the sleep hygiene technique".* | Click on “Fichas de Trabalho” > “Sono-Exercício 1: Diário de Sono” > Enviar comentários > Type text > Send |  |  |  |
|  | Task 13 | *“Para além dos trabalhos de casa, deve monitorizar/medir o progresso terapêutico desta doente desta última semana”.*  *"In addition to homework, you should monitor the therapeutic progress of this patient in this past week"* | Go to “Iniciar”, “Admin” or “Voltar atrás” |  |  |  |
|  | Task 14 |  | Click on “Questionários” |  |  |  |
|  | Task 15 | *“Verifica que não foi administrada nenhuma escala a esta doente e decide fazê-lo. Pf agenda a administração da bateria de testes semanal, estipulando amanhã como a data de início”.*  *“You realize that no scale has been administered and decide to do it. Please schedule the administration of a weekly assessment, stipulating that the start date is tomorrow”.* | Go to “Iniciar” or “Admin” and click on “Utilizadores > Tina Turner” |  |  |  |
|  | Task 16 |  | Click on  “Protocolo de avaliação” > Bateria de testes - semanal |  |  |  |
|  | Task 17 |  | Save the previous procedure |  |  |  |
| E: Conversations | Task 18 | *“Decide enviar um e-mail à sua doente de modo a sublinhar a necessidades desta preencher a bateria de testes prescrita”.*  *Please send an e-mail to this patient underlining the importance of filling-in the prescribed scales.* | Go to “User” or “Iniciar” OR “Admin” > Tina Turner > Mensagens > Nova mensagem |  |  |  |
|  | Task 19 |  | Send an e-mail to Tina Turner |  |  |  |
|  | Task 20 | “*Entretanto recebeu uma resposta desta a pedir-lhe para iniciar uma conversa via chat. Diga por favor “Olá” a Tina Turner”.*  *“In the meantime, the patient replied asking you to start a chat conversation. Please say "Hello" to Tina Turner ".* | Click on “Chat” > “Ir para chat” and type “Olá” |  |  |  |
|  | Task 21 | *“A Tina parece-lhe estar a ter dificuldades em expressar-se por escrito e decide que é melhor falarem face-a-face. Por favor inicie essa conversa”.*  *“Tina seems to be having a hard time expressing herself in writing and you decide that it is better to speak face-to-face. Please start this conversation”.* | Activate the video-conference function and call Tina Turner |  |  |  |
|  | Task 22 | *“Após concluir esta sessão decide atualizar o diário clínico desta doente, escrevendo “Próxima sessão em uma semana”.*  *“After completing this session, you decide to update the patient's clinical diary, writing “Next session within a week”.* | Go to “User” or “Iniciar” OR “Admin” > Tina Turner > Adicionar “nota diário” |  |  |  |
|  | Task 23 | *“Após o registo decide marcar a próxima sessão para daqui a uma semana dando instruções à plataforma para a alertar a si e à Tina Turner 15 minutos antes.”*  *"After registering your notes, you decide to schedule the next session a week from now giving instructions to the platform to alert you and Tina Turner 15 minutes before the onset of the session."* | Click on calendário > Select date on the calendar or click on “Adicionar” and schedule the event and alarm accordingly. |  |  |  |
| **Instructions:**Register the number of**P –** Pass; **F -**Fail; and **A –** Assistances occurrences. **Instructions:**Register the number of**P –** Pass; **F -**Fail; and **A –** Assistances occurrences. A task will only be completed if the condition of the task is true. An error should be counted every time the participant performs an action that does not contribute to task completion.  An assistance is considered every time the participant requests the assistance of the facilitator to perform the task. If the assistance is required because the task was not well explained it should not be considered as assistance in task completion. If the facilitator intervenes because he feels the participants needs help, it should also be accounted for as an assistance. | | | | | | |
