# Supplementary material for: Development and Evaluation of the Usefulness, Usability, and Feasibility of iNNOV Breast Cancer: Mixed Methods Study
Source: JMIR Cancer. 2022 Feb 15;8(1):e33550. doi: 10.2196/33550 (PMC8889471; doi:10.2196/33550)
Supplement: Multimedia Appendix 4 [file cancer_v8i1e33550_app4.docx]

**Multimedia Appendix 4: BCS and MHP Debriefing interview’s scripts**

**Breast Cancer Survivors’ debriefing interview’s script** (in Portuguese followed by a translation to English)

Então, o que achou? (*So, what did you think?*) or Como é que correu? (*How did that go?*)

Como se sentiu ao utilizar a plataforma? (*How did you feel using the platform?)*

Quais foram as principais dificuldades que sentiu? (*What were the main difficulties you experienced?*)

Quão difícil foi ultrapassar essas dificuldades? (*How difficult was it to overcome these difficulties?)*

Que outros problemas identificou na plataforma? (*What other issues did you identify with the platform?*)

Tem alguma sugestão de como poderíamos solucioná-los? (*Do you have any suggestions on how to solve them?*)

Como é que explicaria a uma outra mulher que tenha tido cancro da mama, em que é que consiste esta plataforma? (*How would you explain to another woman who has had breast cancer what this platform consists of?*)

Qual a funcionalidade que achou que seria mais útil para o seu caso? (*Which feature did you think would be most useful for you?)*

Há mais alguma coisa que gostasse que a plataforma permitisse fazer? (*Is there anything else you would like the platform to do or include?*)

Em que circunstâncias consideraria utilizar esta plataforma? *(“Under what circumstances would you consider using this platform?”*)

Espera-se que, durante a fase de tratamento, esta plataforma seja utilizada pelo menos uma vez por semana, durante cerca de uma hora. Considerando as suas outras responsabilidades e compromissos, acha que seria para si possível utilizar esta plataforma? (*It is expected that during the treatment phase this platform will be used at least once a week for about one hour. Given your other responsibilities and commitments, do you think it would be possible for you to use this platform?*)

Acha que precisaria de ter algum tipo de formação para começar a utilizar a plataforma? *(“Do you think you would need some training to start using the platform?”*)

Se sim. Como se deveria organizar essa formação (presencial, online, folheto, etc.)? (If yes. *“How should this training be organized (in person, online, leaflet, etc.)?”*)

Há mais alguma coisa que gostasse de referir, que não tenhamos ainda abordado? *(“Is there anything else you would like to mention that we have not yet addressed?”*)

Gostaria de poder explorar os conteúdos e funcionalidades desta plataforma com mais tempo? *(“Would you like to explore the content and features of this platform for a longer period?”*)

Se sim. Estaria disponível para, durante as próximas duas semanas, experimentar 2 dos módulos de tratamento que desenvolvemos e dar-nos a sua opinião sobre os mesmos? (If so. *“Would you be available for the next two weeks to try out 2 of the treatment modules we have developed and give us your feedback on them?”*)

If participants agree to participate in the home-based test, make sure to provide instructions flyer, credentials to the system and schedule a date/time to collect participants’ feedback.

**Mental Health Professionals’ debriefing interview’s script** (in Portuguese followed by a translation to English)

Então, o que achou? (*So, what did you think?*) or Como é que correu? (*How did that go?*)

Como se sentiu ao utilizar a plataforma? (*How did you feel using the platform?)*

Quais foram as principais dificuldades que sentiu? (*What were the main difficulties you experienced?*)

Quão difícil foi ultrapassar essas dificuldades? (*How difficult was it to overcome these difficulties?)*

Que outros problemas identificou na plataforma? (*What other issues did you identify with the platform?*)

Tem alguma sugestão de como poderíamos solucioná-los? (*Do you have any suggestions on how to solve them?*)

Como é que explicaria a um colega de trabalho, em que é que consiste esta plataforma? (*How would you explain to a patient/client what this platform consists of?*)

Qual a funcionalidade que achou que seria mais útil para o seu trabalho? (*Which feature did you think would be most useful for your work?)*

Há mais alguma coisa que gostasse que a plataforma permitisse fazer? (*Is there anything else you would like the platform to do or include?*)

Em que circunstâncias consideraria utilizar esta plataforma? *(“Under what circumstances would you consider using this platform?”*)

Que dificuldades antecipa que pudessem existir, se este sistema fosse introduzido no seu contexto de trabalho? (*What difficulties do you anticipate might exist if this system were introduced into your work context?*)

Acha que precisaria de ter algum tipo de formação para começar a utilizar a plataforma? *(“Do you think you would need some training to start using the platform?”*)

Se sim. Como se deveria organizar essa formação (presencial, online, folheto, etc.)? (If yes. *“How should this training be organized (in person, online, leaflet, etc.)?”*)

Há mais alguma coisa que gostasse de referir, que não tenhamos ainda abordado? *(“Is there anything else you would like to mention that we have not yet addressed?”*)
