# Supplementary material for: Development and Evaluation of the Usefulness, Usability, and Feasibility of iNNOV Breast Cancer: Mixed Methods Study
Source: JMIR Cancer. 2022 Feb 15;8(1):e33550. doi: 10.2196/33550 (PMC8889471; doi:10.2196/33550)
Supplement: Multimedia Appendix 5 [file cancer_v8i1e33550_app5.docx]

**Multimedia Appendix 5: Field trial protocol**

Following the post-test interview, BCS should be asked to continue using the platform during two weeks, and complete one intervention module. This specific test aims at assessing the usefulness of the platform and its content. The moderator should introduce the participant to the following scenario, contextualizing the execution of test tasks:

Scenario: BCS (optional): *“Após se ter registado e aprendido a navegar o website, decide iniciar o tratamento e durante duas semanas, ler o módulo [a selecionar pela participante]”.*

*“After registering and learning how to navigate the website, you decide to start the treatment and for two weeks, read the module [to be selected by the participant]”.*

This scenario endorses the following tasks (see Table 1):

| **Table 1.** Field trial tasks for BCS | |
| --- | --- |
|  | **Tasks** |
| **1** | Change password to access INNOVBC |
| **2** | Reading the content of the modules |
| **3** | Completing the module’s exercises |
| **4** | Assessing and rating the module |
