# Supplementary material for: Development and Evaluation of the Usefulness, Usability, and Feasibility of iNNOV Breast Cancer: Mixed Methods Study
Source: JMIR Cancer. 2022 Feb 15;8(1):e33550. doi: 10.2196/33550 (PMC8889471; doi:10.2196/33550)
Supplement: Multimedia Appendix 6 [file cancer_v8i1e33550_app6.docx]

**Multimedia Appendix 6: Usefulness and feasibility questionnaire**

**Parabéns! *Congratulations!***

**Chegou ao fim do primeiro módulo de tratamento/*You have reached the end of the first treatment module.***

1. Top of Form

**1. Qual a sua opinião acerca da utilidade dos conteúdos incluídos neste módulo de tratamento?**

Top of Form

***What is your opinion about the usefulness of the contents included in this treatment module?***

Selecione a sua resposta considerando a escala apresentada em baixo, em que 1=Totalmente inútil e 5= Extremamente útil.

*Select your answer considering the scale provided below, where 1=Totally useless and 5=Extremely useful.*

Totalmente inúteis/*Totally useles*s 1  2  3  4  5 Extremamente úteis/*Extremely useful*

**2. Qual o grau de adequação dos conteúdos deste módulo às suas dificuldades e necessidades?**
***How well do the contents of this module suit your difficulties and needs?***

Selecione a sua resposta considerando a escala apresentada em baixo, em que 1=Totalmente inadequado e 5=Extremamente adequado.

*Select your answer considering the scale provided below, where 1=Totally inadequate and 5=Extremely adequate.*

Totalmente inúteis/*Totally inadequate* 1  2  3  4  5 Extremamente adequados/*Extremely adequate*

**3. Quantos minutos demorou a completar este modulo de tratamento?**
***How many minutes did it take to complete this treatment module?***

**4. Recomendaria este módulo de tratamento a um amigo ou familiar que se encontrasse numa situação semelhante à sua? (**Sim/ Não)

***Would you recommend this treatment module to a friend or family member who is in a similar situation to yours?***

**5. De um 1 a 5, como classificaria este modulo de tratamento?**

***From 1 to 5, how would you classify this treatment module?***

☆ ☆ ☆ ☆ ☆

**Outros comentários/*Other comments*:**

Bottom of Form
