# Supplementary material for: Development and Evaluation of the Usefulness, Usability, and Feasibility of iNNOV Breast Cancer: Mixed Methods Study
Source: JMIR Cancer. 2022 Feb 15;8(1):e33550. doi: 10.2196/33550 (PMC8889471; doi:10.2196/33550)
Supplement: Multimedia Appendix 7 [file cancer_v8i1e33550_app7.docx]

| **Multimedia Appendix 7: Identified usability issues** | | | | | |
| --- | --- | --- | --- | --- | --- |
| **Platform section or feature** | **Issue category** | **Issue description** | **Source of information** | **Example quote:** | **Action taken or planned** |
| Design | Effective information presentation | Colour scheme not informative | Feedback | “I found it a little monotonous, everything looked the same (…) and that confused me. Maybe using more colour and highlighting some parts (…) it would be easier to understand how the materials are organized…” (BCS6) | Make additional colour tests |
| Design | Effective information presentation | Text not readable | Feedback | “I need the letters to be bigger” (BCS4) | Increase the font size/Include less information in each screen |
| Design | Effective information presentation/Efficient interaction | Low use of images and icons | Feedback | “I didn't find it very appealing. (...) More appealing would be... like, having more figures, not being so professional. I think it's very professional. Sorry for saying this... But it felt like filling-in a questionnaire... Not because of your instructions but, because of how it looks” (BCS8) | Further develop the programs’ iconography/Convert some features and information into icons and images |
| Design | Cognitive load | Busy pages with long text or features | Feedback | You can’t have too much information in one page (…) it should be like… materials for children with autism (…) the information must be very clear, concise and short, otherwise people get disorganized” (BCS6) | Simplify the design/Reduce the content and features presented in each screen/Create supplementary information feature |
| Navigation | Naturalness/Efficient interaction | When changing pages or clicking in the expandable text, pages do not show the new content or features making users unaware of the additional text and features that are available | Observation, Errors, assistances, feedback | "The screen should move or show that something happened, I wasn't understanding that new buttons opened" (MHP6) | Automatically centre the page to show the new content and features being displayed/Whenever possible show all information in one central screen, to avoid scrolling/ Program so when a section of text is expanded, other sections are compressed |
| Navigation | Consistency | Return button sometimes leads to the homepage and not the page seen previously | Observation | Not applicable | Fix bug |
| Login | Cognitive load | Login and recover password menus are equally emphasized leading participants to believe an e-mail should be entered to access the platform | Observation, Errors | Not applicable | Reposition the recover password menu and make it available only at login failure |
| Login | Preventing errors | Typographic error at login | Observation, Errors | Not applicable | Add show password functionality |
| Login | Preventing errors | Error message does not discriminate the error type | Observation | Not applicable | Detail type of error being performed |
| Homepage | Naturalness | Home button linked to studies’ public pages, confuses users, and leads to a try and error behaviour when trying to access the homepage | Observation, Errors | Not applicable | Link home button to the homepage and add public pages shortcut icon to the toolbar |
| Homepage | Efficient interaction/Cognitive load | Organization of the homepage in columns makes users opt for working with only one side of the page, frequently with the functionalities side, ignoring the notifications area | Observation, Errors, Assistances, Feedback | “There were 2 beginnings… a very large one and then the other that was already inside the platform… And I was a little confused with which one to use to actually begin using the platform… on the left or on the right? (…) So I chose the one with the bigger font” (BCS3)  “I think it would be more intuitive if on the dashboard, on the page, when we log in, if there were those little figures for people to click on right away… Like an app, isn't it? We get there and we have those images that make us associate the image with a function…” (BCS8) | Display all features in the middle of the page/Create visual centralized dashboard/Make notifications area more visible and adopt a design similar to the apps frequently used by end-users (e.g., Pinterest, LinkedIn, WhatsApp, Facebook, Facetime, OLX, etc.) |
| Homepage | Cognitive load | The welcome message is too long and permanent, providing orientations on where to click and interrupting users natural course of action | Feedback | "This is too much text and it doesn’t contain important information (…) It distracts me" (MHP7)  “The fact that it says here to click on the start button ends up conditioning my intuitive behaviour” (BCS5) | Delete the instructions provided in the welcome message/ Make available to first visitors a virtual tour highlighting the platform main features |
| Homepage menu | Efficient interaction/ Forgiveness and feedback | Users struggle to understand what is inside each menu function and expect to be able to preview what is inside each module | Feedback | “I think a constant bar with all the menus would be easier in terms of orientation (…)” (BCS1)  “Having an icon or sort of preview of the next topics… modules would facilitate the use of the platform” (BCS3) | Create centralized dashboard with labels and preview options/Create task bar with treatment modules always available |
| Notifications | Effective information presentation/Naturalness | Selected colour scheme does not communicate priority, making users ignore it | Observation, Errors, assistances, feedback | “I think it should be a stronger colour because as is, it doesn’t call may attention… It doesn’t look like an alert (…) maybe a more appealing colour like red, so I could be reminded to go there (…) and maybe be at a sequential position, not in parallel to the other options” (BCS2) | Change notifications colour to make it more noticeable/Create notifications centre |
| Notifications | Effective information presentation | Hyperlink to access the notifications’ text not noticeable and interpreted as a preview of the message inhibiting users to click on it and access the whole content | Observation, Errors, assistances, feedback | “This way of presenting the information seems like an appointment… already showing the content of the message and not something that you should click on” (BCS5) | Highlight links embedded in notifications/Add “see notification” instruction/Expand clickable area |
| Users hub | Effective use of language | Users label leads professionals to assume it refers to other healthcare professionals working with the program that are online and not clients/participants; Tick option interpreted as the pathway to access patients’ files. | Observation, Errors, assistances, feedback | “This should have a list of patients and a way to access the patients’ files, but where is it?” (MHP2)    "The designation users confuses me, it is too impersonal" (MHP6)  “When I saw users, I thought it meant me and the other professionals using the platform (…) off course the clients are users of the platform but it’s not a designation I am used to” (MHP10)  "This should have a text popping up with the description of the function of the button" (MH7) | Change designation to patients, clients or participants depending on the context of use/ Add labels explaining the purpose of each feature. |
| User hub | Efficient interaction/Cognitive load | Therapists get confused with the expandable details, configurations and log options that are available and try opening all folders to complete a given task. They expect to find an overview of the patient status at each given time. Main actions like registering data in patients’ journals are hidden and dependent of scrolls and, therefore, not noticed. Therapists keep clicking on users’ e-mail expecting to be able to send a message to the participant | Observation, Errors, assistances, feedback | "It's boring to have to return to users every time" (MHP11)  "This is not intuitive, clicking on the name should be enough to send an e-mail to the participant" (MHP5) | Provide data visualization dashboards summarizing graphical information about the patient/Create configurations binder to include administrative information/ Create patient journal binder/Insert labels explaining the intent of each binder/ Add link to conversations section/Make conversations always available/Create a conversations binder/ Simplify layout/ Create centralized dashboard displaying all features in the middle of the page to avoid scrolls/Show main actions option first |
| User hub | Naturalness/Efficient interaction | Therapists cannot see the patient journal link, looking for that option on the clinical data binder or trying to edit the logs. Users expect that all notes and patient’s actions to be chronologically organized is a prominent area of the platform. Main function is presented below the confifuration information about the patient and not seen by users | Observations, Errors, Assistances, Feedback | "Where's Frida's clinical file?" (...) This is in a very bad location, completely undervalued, this should be and important and salient binder". (MHP6) | Relabel “notas do diário” to “Registos clínicos” /Create a binder for the patient journal and integrate logs and communication exchanges chronologically in an e-mail expandable feature |
| User hub | Effective use of language | The word connection/connected to is interpreted as the patient being online and being allowed to see therapists’ notes | Feedback |  | Change designation to Terapeuta responsável: xxx foi atribuído a xxx |
| User hub | Efficient interaction | Divide between the conversations menu and the treatment section either viewed by therapist or patients, breaks users flow while using the program leading them to adopt a try and error behaviour, while searching for the correct button to message the therapist/provide feedback to the patient. | Observation, Errors, assistances, feedback | "The transition from the patient file to conversations to is not linear, I get there but is by trying several buttons" (MHP11); "I'm lost, it's hard to get here" (MHP10)  “Imagine I am reading something, and I am not understanding it well or I want to tell what is happening to me to the therapist… Just the fact that I have to go to another page and look for it [conversations section] creates a huge mess because you don’t know where to go and when you get there you don’t remember anymore where you were at… So… I think the chat should be always available… at the bottom or somewhere else” (BCS7) | Make conversations menu always available (i.e, not changing the user view)/or add a folder inside the User hub linking to the conversations section and logging the exchanged communications between therapists and participants. |
| User hub | Naturalness/Customizability/Flexibility | The user hub is sometimes interpreted as a therapists’ main toolbar and not a menu to manage each participant | Observation, Errors, assistances, feedback |  | Make clear divide between the user hub and admin functions |
| User hub | Effective information presentation | Message to select a given questionnaire is not understood and therapists believe the folder is empty | Observation, Errors, Assistances |  | Display answered questionnaires in a log form. |
| User hub | Consistency | Envelope next to assigned questionnaires interpreted as messaging system and not a log of messages sent. The envelope is shown regardless of the option to e-mail participant is selected or not. | Observation, Errors, Assistances |  | Link to conversations and log alert e-mails as messages exchanged between client and therapist. |
| Conversations | Cognitive load/Naturalness | Conversations layout including too much information, confuses users, hindering tasks completion | Observation, feedback | “We are all very familiar with WhatsApp, messenger, etc. which are much simpler platforms than this one, everything is simple and intuitive... This platform ends up being a little bit complicated because there’s so much in here… that we get lost, so… I believe using the same layout as those platforms would be more effective…” (BCS7) | Simplify layout, adding temporary labels to the main features and eliminating accessory information/ Design with most used platform as reference/Rename tópico to assunto in the conversations log. |
| Conversations | Efficient interaction | The chat editable area is often hidden, requiring scrolls and participants don’t understand where to write a message. Users often click on the chat, but not the editable box. | Observation, Errors, assistances, feedback |  | Centre chat in the screen/ eliminating scrolls/Expand clickable area/ Add “Write a message” in the editable text box |
| Conversations | Efficient interaction | Users look for a direct link to the treatment modules/worksheets inside therapists’ messages | Observation, feedback | “If I click in the therapist's message, does it open this information?” (BCS3) | Add the possibility of adding hyperlinks to other sections of the platform in the messages |
| Treatment modules | Effective use of language/Consistency | “Intervention modules” label is not understood and is used in different sections of the platform for different purposes, leading to different views/results and leading users to try to complete the tasks using a trial and error approach. | Observation, Errors, assistances, feedback | Iterations on the designation treatment modules>treatment>Intervention>Intervention modules    "Reaching the modules is not very intuitive it took me a while (…) considering that some people might have a lower education level… I believe I would call this content…informative content…" (BCS3)  "Different functions with the same labels confuse me, it's not clear to me how to do this. Where is Frida?" (MHP7) | Use different labels in each section/always lead the user to a unique menu or view when labels are repeated |
| Treatment modules | Customizability/Consistency | The treatment modules link in the homepage leads to the content of the whole program as seen by patients and confuses therapists, as they do not understand what has been assigned to whom and how to edit the content. | Observation, Errors, assistances |  | Always link the same link/designation to the same feature/Show outline view first to therapists (as in admin), adding preview and editing functionalities on the top menu/Make all sections and designations consistent. |
| Treatment modules | Forgiveness and Feedback | It is not clear to therapists which treatment modules are assigned to each patient and when changes are performed if those changes were actually performed. | Observation, Errors, assistances |  | Reformulate text to make clear what the assigned modules are and what are the ones available to be assigned. Reformulate text “Módulos de intervenção para teste”, to “Módulos atribuídos a:”. Disable save button when no further changes have been performed |
| Treatment modules | Naturalness/Efficient interaction | The main function of assigning a treatment protocol/plan is presented below a list of treatment modules and not seen by users |  | "I'm doing this in a try and error basis, I’m not sure I would be able to remember this if I had to do it again" (MHP8). | Simplify layout/ Create centralized dashboard displaying all features in the middle of the page to avoid scrolls/Show main actions option first/on the top. |
| Treatment modules | Effective use of language/Forgiveness and Feedback | Users hesitate to click on the save button when trying to send an e-mail informing that a treatment module has been assigned because they don’t know if the message will be sent/was sent. After prescribing a module it is not possible to confirm if a notification was sent. This option is only available when a treatment protocol in assigned. | Feedback | "Save button should be send, I'm not sure she was notified" (MHP6) | Change save designation to send/Automatic reminder and alerts should be logged in the conversations log. |
| Treatment modules | Consistency | Treatment modules shortcut in the top task bar not working and leading to homepage in patients view | Observation |  | Resolve bug and link menu option to treatment modules section |
| Treatment modules | Effective information presentation | The active section/page inside the treatment modules is not salient enough making users doubt which section they are reading at each time | Observation, Feedback | “Everything is presented with the same colour, that doesn't… for eg, we are here [treatment module outline] but if this were in a different colour, I could see which section of the module I am actually using…” (BCS6) | Change the colour of the binder when text is being read, to make it more salient and communicate to users which page they are reading |
| Treatment modules | Cognitive load/Efficient interaction | Numerous sections and long text inhibit some users of interacting with the modules/ An integration and simultaneous use of various modules at the same time seems to be expected | Feedback | “I like using links and I think the minimal should be displayed in each page… And a unique screen should be enough… Imagine like… a sort of PowerPoint… I would click on different thigs, but always in the same main page… then if there are articles to read or something, I am aware of that, but always using the same page” (BCS7)  “When I started reading the relaxation part, it felt like I was reading an article (…) I think I would organize it like this… you would have several squares… icons… and then people would explore it… you know? Now I am going to relax, and you would have the audios, etc… There wouldn’t be a list on the side, because it looks like… it is very formal, professional… And if there were the little icons people would… oh that’s funny, I will click here” (BCS8)  "The number of sections and the length of the text is too much. It makes me give up and not read or use this" (BCS3) | Balance the length of the text within sections/Create supplementary readings section for users that are interested in reading further information/Assess pertinence of creating a centralized menu option/Adopt a more informal and cheerful tone when writing content and in the design/Create a personalized shortcuts toolbar for users to access most used features and content |
| Treatment modules | Effective information presentation/Customizability/Flexibility | The content should be displayed in various formats like audio, video, and written files | Feedback | “Audios and videos… If the person doesn’t want to read or has a lot of difficulties in understanding, that could help” (BCS8) | Diversify/add alternative and inclusive ways of displaying content |
| Treatment modules | Customizability/Flexibility | Audio files are not diversified and stop when other notifications are displayed on mobile phones | Feedback | “When I performed the relaxation, I was interrupted by notifications on the phone (…) but I really liked the audio” (BCS3)  “I would prefer a woman’s voice… I guess I would identify more easily (…) and then adding some background sound would help… like the sea sound…” (BCS6) | Include alternative recordings/Develop app version |
| Treatment modules | Effective information presentation | Exercises examples imbedded in the modules and worksheets are often not interpreted as examples, but as instructions | Feedback | "I'm lost already, did I advance too much?" (BCS4)  "The example induces in error; it seems to establish a limit of hours that can be registered or to ask me to register what I did at that time" (BCS2) | Simplify examples and use specific and transversal design to present examples |
| Treatment modules | Cognitive load/ Efficient interaction | Users find tables too complex to fill-in and do not understand tables headings “Morning 1 or day 1” | Assistances, Feedback | It should have an option to enter the date and then the system would label it as register 1, 2, 3, etc… automatically. This shouldn't be a table, it should be a simple field to complete and then the psychologists would see the table. If it's important to the patient to see the progress, a different graphical approach should be used, easier to understand than this complex table. (BCS11) | Diaries should be completed using a plain text box and automatically transformed into a table in the therapists’ view and in graphical information for the patients to see their progress using a data visualization dashboards. |
| Treatment modules | Efficient interaction | Users do not always see the printing option on the top of the page, especially when they scrolled the text, and try to use keyboard shortcuts to print the whole module, but only the page they are at is available for printing using that pathway. | Observations, Errors, Assistances, Feedback | "This printing icon should always be visible"” (BCS1) | Pin a toolbar including transversal functionalities, so the printer and “see whole module feature” are always seen and available to users. |
| Worksheets | Effective use of language | “Fichas de trabalho” is not understood and users are not comfortable with the “homework designation”. Most common suggestion was tasks, but some therapists interpreted tasks as their own tasks and not the one to be assigned to patients. Some patients think tasks and agenda are similar functions. | Feedback | Iterations on the designation worksheets (Fichas de trabalho)>tasks (Tarefas)  “I think the terms used weren’t completely obvious… It was hard for me to understand how to find the patient, how to consult the things that she had performed, the tasks I had assigned to her (…) It confused me because those were not the terms I use in my practice (…) I believe something closer to what I use daily, closer to the platforms we have there [at the hospital] would be easier (…) like list of patients… clinical file…prescriptions… results…” (MHP12) | Use different labels in each section/always lead the user to the same menu when labels are repeated/Possibly create a therapists section or toolbar displaying their pending tasks and actions. |
| Worksheets | Customizability/Flexibility | Users expect to find a visual presentation of their treatment progress | Feedback | “Both for the therapists and the patients… taking into account that there are a lot of items to be filled in the platform, I believe having a summary, and overview… that helped me realize my progress… maybe presented like a graph, not only in writing… and something beautiful to see… would make me feel, you know?... Wow, I managed to get here today… Imagine, all the questionnaires… Having a certain score and then at the end it’s done! You know? As children have at school, the green, yellow and red stars… Anything like that… Maybe I’m being childish… but for those who are at this stage… Positive reinforcement is needed…” (BCS11) | Develop data visualization dashboards |
| Agenda | Efficient interaction | Users click on the date, but the add event page is not shown, because they click outside the clickable area. |  | "This is frustrating" (MHP7) | Expand clickable area. |
| Agenda | Effective use of language | The time field is labelled as date, confusing users. | Feedback | “In hours it should state hour and not date" (BCS9) | Change label to “Hora (hh:mm)” |
